# Development and validation of a pediatric model predicting trauma-related mortality

**DOI:** 10.1186/s12887-023-04437-9

**Published:** 2023-12-18

**Authors:** Mary Evans, Karthik Rajasekaran, Anish Murala, Alvaro Moreira

**Affiliations:** 1 McGovern Medical School, 6431 Fannin St, Houston, TX 77030 USA; 2 Department of Otorhinolaryngology, Penn Medicine, Hospital of the University of Pennsylvania, 800 Walnut St, 18Th Floor, Philadelphia, PA 19107 USA; 3 Texas A&M University, Administration Building, 400 Bizzell St, College Station, TX 77843 USA; 4 Neonatology Regenerative and Precision Medicine Laboratory, University of Texas Health Science Center at San Antonio, San Antonio, TX USA

**Keywords:** Pediatric mortality, Trauma prediction, TRAGIC+ Model

## Abstract

**Objectives:**

To develop a prediction model of mortality in pediatric trauma-based injuries. Our secondary objective was to transform this model into a translational tool for clinical use.

**Study design:**

A retrospective cohort study of children ≤ 18 years was derived from the National Trauma Data Bank between the years of 2007 to 2015. The goal was to identify clinical or physiologic variables that would serve as predictors for pediatric death. Data was split into a development cohort (80%) to build the model and then tested in an internal validation cohort (20%) and a temporal cohort. The area under the receiver operating characteristic curve (AUC) was assessed for the new model.

**Results:**

In 693,192 children, the mortality rate was 1.4% (*n* = 9,785). Most subjects were male (67%), White (65%), and incurred an unintentional injury (92%). The proposed model had an AUC of 96.4% (95% CI: 95.9%-96.9%). In contrast, the Injury Severity Score yielded an AUC of 92.9% (95% CI: 92.2%-93.6%), while the Revised Trauma Score resulted in an AUC of 95.0% (95% CI: 94.4%-95.6%).

**Conclusion:**

The TRAGIC + Model (Temperature, Race, Age, GCS, Injury Type, Cardiac-systolic blood pressure + Mechanism of Injury and Sex) is a new pediatric mortality prediction model that leverages variables easily obtained upon trauma admission.

**Supplementary Information:**

The online version contains supplementary material available at 10.1186/s12887-023-04437-9.

## Introduction

Despite medical advances, trauma continues to be the leading cause of mortality and acquired disability in children. In 2015 alone, there were more than 11,000 deaths and over 8 million nonfatal injuries caused by trauma to children between the ages of 1 and 19 [1]. Of children who die from traumatic injuries, most die within the first 24 h upon hospital arrival [2]. Therefore, due to its high prevalence, up-to-date information regarding pediatric trauma is continuously being produced and brought to the attention of clinicians. For instance, current literature regarding trauma-related pediatric injury shows that firearm injuries account for more than 25% of all unintentional deaths among children. More alarming, this rate now puts firearm injuries as the number one cause of death for children in the United States, surpassing motor vehicle accidents [3, 4].

Several prediction models for pediatric mortality outcomes have been developed and can assist in decision making [5–9]. Some of these scoring systems are for triage prior to hospital admission, while others assess injury severity or mortality outcomes in manners that are complex, performed retrospectively, or time-consuming [5]. The Injury Severity Score (ISS) is a commonly used trauma scoring system that has been validated in the pediatric population. Although the ISS associates well with trauma-related mortality, it does have some setbacks. For instance, it requires specialized training and can only be done for research purposes as the calculation is performed retrospectively [6]. The Revised Trauma Score (RTS) is another example of a well-established and widely used injury scoring system. This tool leverages respiratory rate, Glasgow Coma Scale (GCS), and systolic blood pressure into consideration, but fails to take into account any non-physiologic variables that may also be central in determining survival. Finally, the pediatric trauma ‘BIG’ score uses base deficit, International Normalized Ratio (INR), and GCS as predictive variables. Despite the novelty, it was developed in a small military population which puts into question the generalizability of the model, as most patients succumbed to a blast injury [9–11]. More importantly, many of these prediction models were built before guidelines for methodologic rigor were established, (e.g. TRIPOD statement).

The purpose of this study was to create a pragmatic mortality prediction model for children treated in a U.S. trauma center. We included all-cause trauma-related injuries and relied on using readily available clinical/physiologic variables. Such a prediction model would quickly provide impactful information to clinicians who can better make treatment decisions and more accurately inform patients’ families. Our secondary objective was to transform this prediction model into a web-based dynamic nomogram that can quantify mortality risk.

## Methods

### Data source

This is retrospective cohort study of patient data derived from the National Trauma Data Bank (NTDB) from 2007 to 2015. The NTDB is a nationally representative sample of individuals cared for in > 900 trauma centers in the United States.

Details regarding the NTDB can be viewed at the “About Trauma” subsection of the NTDB website [12]. Data regarding the classification of predictors used in this study per the NTDB can be viewed on the NTDB’s data dictionary [13]. This study adhered to the guidelines set by the Transparent Reporting of a Multivariable Prediction Model for Individual Prognosis or Diagnosis (TRIPOD) [14] (Supplementary File 1).The data is de-identified and therefore did not require institutional review board approval.

### Patient cohort and objectives

We selected all children (≤ 18 years) presenting to any trauma center irrespective of injury. We excluded patients that did not have information pertaining to ED or hospital disposition or who had missing age. Children who died prior to ED arrival were not included. The primary outcome was death within the ED or during hospitalization.

Our objective was to identify variables that could serve as early markers of death in children with a traumatic injury. As such, the predictors included patient age, gender, race, Glasgow coma scale (GCS), mechanism of injury, and physiologic data on arrival to the ED. Categories for mechanisms of injury and intent in our analysis were based on the standard nomenclature used by the National Trauma Bank (NTDB). The only mechanisms of injury that had to be altered were ‘Hot object/substance’ and ‘Transport, other’. These were added to ‘Other specified and classifiable’ as there were so few of these mechanisms in the dataset. The complete list can be seen in Supplementary Files 2a, b, 3a and b. All physiologic data was obtained at the time of arrival to the emergency department.

Predictive mean matching and simple bootstrapping methods were used to impute missing data [15]. Respectively, Supplementary File 2a and b represent the imputed and non-imputed study characteristics by death, respectively while Supplementary Files 3a and b represent the imputed and non-imputed study characteristics by race, respectively. Supplementary File 4 provides specifics regarding percent of missing data and a comparison of missing data versus imputed data.

### Development and internal validation cohort (2007–2014)

**Fig. 1:**
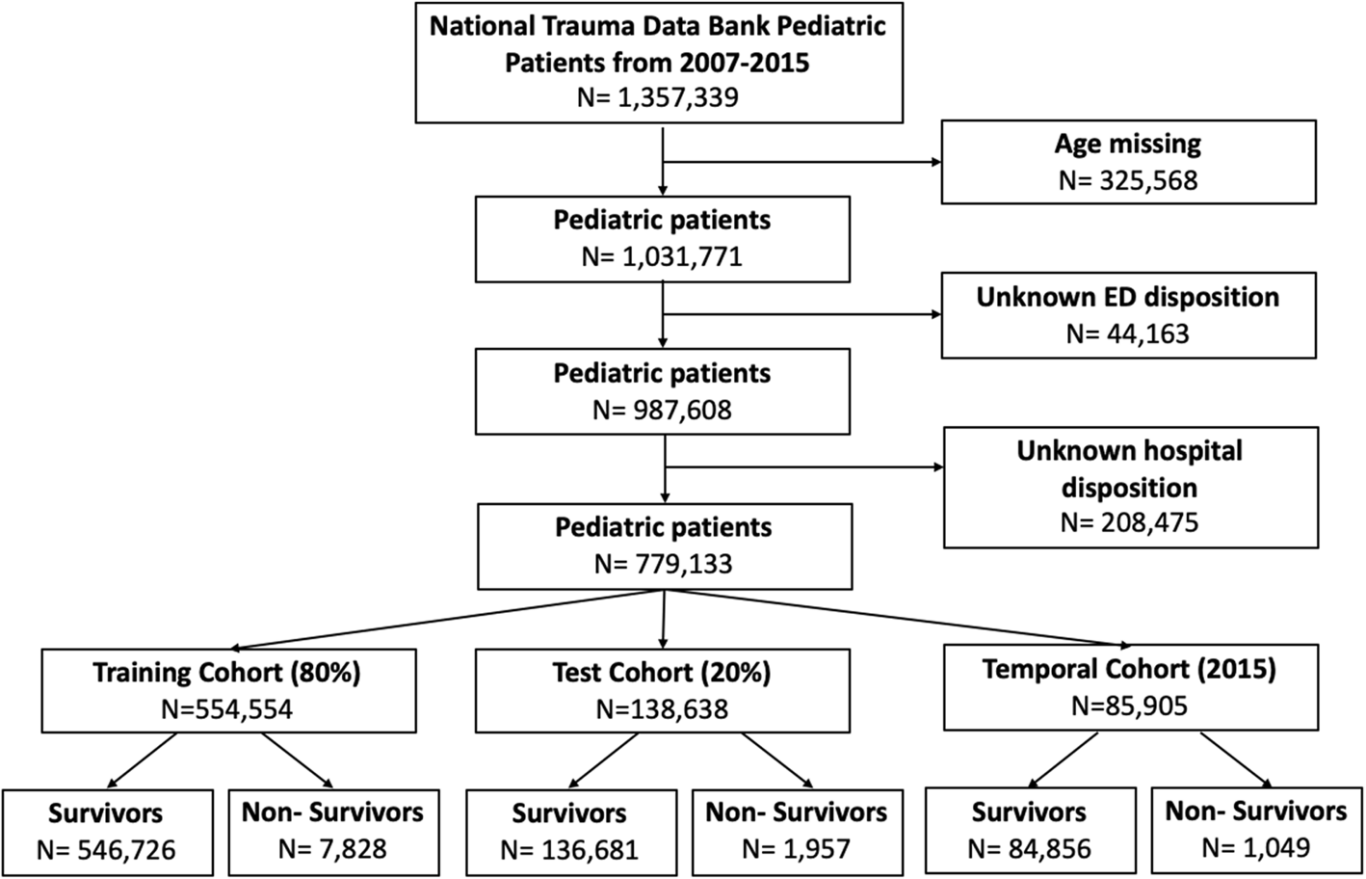
Study outline

Individuals were randomly split into a training set (80%) and a test set (20%). In this way, we could evaluate the predictive ability of the new model in a new set of data to confirm validity. Figure 1 illustrates the study flow chart.

### Temporal validation cohort (2015)

All pediatric patients from the year 2015 served as the temporal validation. Reasoning to have this year as a second validation cohort was to determine whether predictors from over a decade ago still had clinical relevance in a newer cohort of patients that more closely resembled the rapidly evolving trauma management.

### Statistical analysis

Descriptive statistics were tabulated according to survivors and non-survivors. Continuous variables were presented as median with interquartile range (IQR) and analyzed by Wilcoxon rank-sum test. Categorical data were reported as a number with percent and analyzed by the Chi-square test. A *p* value < 5% denoted statistical significance. All analyses were performed with R statistical software version 4.1.0.

Variables found to be significant on univariate analysis were included in a multivariable backward stepwise logistic regression model with a 0.10 revised threshold of test. Variable inflation factors < 4 were kept in the model to minimize collinearity. In the test sets (e.g. internal validation cohort and temporal 2015 cohort), we measured the predictive performance of our model by computing the area under the receiver operating characteristic curve (ROC) and Youden’s index to identify the optimal sensitivity and specificity [16]. The ROC of the new model was statistically compared to the ROC of the RTS and ISS using DeLong’s test [17]. The numeric value of the ISS was reported in the NTDB and was used without any changes to calculate the predictive ability. To create the RTS model, we used the following equation:

$$\mathrm{RTS }= (0.9368\ *\ \mathrm{ GCS\ Value}) + (0.7326\ *\ \mathrm{ systolic\ blood\ pressure}) + (0.2908\ *\ \mathrm{ respiratory\ rate}).$$

As recommended by the TRIPOD statement, calibration curves were conducted in the test set to assess the accuracy between estimated and observed number of outcome events for the prediction model [18]. To conclude, we translated our prediction model into a dynamic nomogram. This web-based application improves the translation of our work into an interactive, user-friendly graphical interface built in RShiny [19].

## Results

### Patient characteristics

Table 1 summarizes the overall study cohort of 693,192 pediatric patients. The majority of the subjects were male (67%), White (66%), and had an unintentional injury (93%). The median age for our subjects was 12 years old [IQR, 6,16] with a median GCS of 15 [IQR, 3,15]. The overall mortality rate was 1.4% (*n* = 9,785). Compared to survivors, children who died were older, had a lower GCS and systolic blood pressure, and were more likely to be a victim of an assault secondary to firearm injury. Mechanisms of injury with the highest mortality rates were firearm (16%), and motor vehicle trauma of occupant (45%), and cut/pierce (1.7%). Other overall study population characteristics can be seen in Supplementary Files 2a, b, 3a, and b.

**Table 1:** NTDB patient characteristics in the development cohort

Variable	Overall	Train data (80%)		Test data (20%)	
	*N* = 693,192	Survivors *N* = 546,726	Non- Survivors *N* = 7,828	Survivors *N* = 136,684	Non- Survivors *N* = 1,957
Age					
0-4y	138,881	109,426 (20%)	1,463 (19%)	27,388 (20%)	361 (18%)
5-9y	148,144	118,164 (22%)	721 (9.2%)	29,448 (22%)	183 (9.4%)
10-14y	157,377	124,823 (23%)	1,169 (15%)	31,071 (23%)	297 (15%)
15-18y	248,790	194,313 (36%)	4,475 (57%)	48,777 (36%)	1,116 (57%)
Gender					
Female	228,179	180,075 (33%)	2,327 (30%)	45,129 (33%)	585 (30%)
Male	465,013	366,651 (67%)	5,501 (70%)	91,555 (67%)	1,372 (70%)
Glasgow coma scale					
Mild (13–15)	638,090	509,687 (93%)	585 (7.5%)	127,559 (93%)	180 (9.2%)
Moderate (9–12)	13,845	10,965 (2.0%)	174 (2.2%)	2,682 (2.0%)	45 (2.3%)
Severe (3–8)	41,257	26,074 (4.8%)	7,069 (90%)	6,443 (4.7%)	1,732 (89%)
Physiologic Measures					
SBP	123 (112, 135)	123 (112, 135)	108 (78, 133)	123 (112, 135)	110 (81, 136)
Pulse	100 (85, 116)	100 (85, 116)	107 (75, 134)	100 (85, 116)	108 (74, 136)
RR	20 (18, 24)	20 (18, 24)	16 (0, 21)	20 (18, 24)	16 (0, 21)
Temp (C)	36.7 (36.3, 37)	36.7 (36.3, 37)	36 (35, 36.7)	36.3 (36.3, 37)	36 (35, 36.7)
Race					
White	453,239	358,319 (66%)	4,359 (56%)	89,415 (65%)	1,146 (59%)
Black/ African American	123,332	96,763 (18%)	2,052 (26%)	24,029 (18%)	488 (25%)
Asian	12,191	9,612 (1.8%)	130 (1.7%)	2,423 (1.8%)	26 (1.3%)
Other	104,445	82,044 (15%)	1,287 (16%)	20,817 (15%)	297 (15%)
Mechanism					
Fall	174,992	139,533 (26%)	311 (4.0%)	35,070 (26%)	78 (4.0%)
Natural/ environmental	5,800	4,594 (0.9%)	21 (0.3%)	1,179 (0.9%)	6 (0.4%)
Cut/Pierce	22,627	17,991 (3.3%)	135 (1.7%)	4,469 (3.2%)	32 (1.6%)
Fire/Flame	5,662	4,438 (0.8%)	87 (1.1%)	1,114 (0.8%)	23 (1.2%)
Firearm	21,311	15,615 (2.9%)	1,357 (17%)	4,025 (2.9%)	314 (16%)
Hot object/ Substance	9,433	7,568 (1.4%)	8 (< 0.1%)	1,855 (1.4%)	2 (0.1%)
Machinery	2,335	1,848 (0.3%)	6 (< 0.1%)	478 (0.3%)	3 (0.2%)
MVT Motorcyclist	9,549	7,535 (1.4%)	139 (1.8%)	1,842 (1.3%)	33 (1.7%)
MVT Occupant	244,295	191,853 (35%)	3,460 (44%)	48,111 (35%)	871 (45%)
MVT Pedal Cyclist	10,859	8,505 (1.6%)	176 (2.2%)	2,130 (1.6%)	48 (2.5%)
MVT Pedestrian	33,574	26,141 (4.8%)	797 (10%)	6,429 (4.7%)	207 (11%)
Overexertion	1,553	1,256 (0.2%)	0 (0%)	297 (0.2%)	0 (0%)
Pedal cyclist, Other	28,949	23,242 (4.3%)	34 (0.4%)	5,660 (4.1%)	13 (0.7%)
Pedestrian, Other	3,248	2,543 (0.5%)	57 (0.7%)	632 (0.5%)	16 (0.8%)
Struck by, Against	52,151	41,402 (7.6%)	219 (2.8%)	10,471 (7.7%)	59 (3.0%)
Transport, Other	40,169	31,950 (5.8%)	267 (3.4%)	7,878 (5.8%)	74 (3.8%)
**Injury Severity Score (ISS)**	5 (4,9)	5 (4,9)	29 (25, 38)	5 (4,9)	29 (25, 38)
**Revised Trauma Score (RTS)**	9.52 (9.52, 9.52)	9.52 (9.52, 9.52)	5.47 (4.45, 5.76)	9.52 (9.52, 9.52)	5.76 (4.89, 5.76)

*MVT* Motor Vehicle Trauma, *RR* Respiratory rate, *SBP* Systolic blood pressure

The temporal data included 85,905 children and can be seen in Supplementary File 5a and b. Children who died had a higher ISS (29 vs. 5), lower GCS (3 vs. 15), and more likely to have had a firearm injury (11% vs. 2.2%). Children had a median age of 15 compared to 11 in the non-survivors versus survivors, respectively. The median RTS was 5.76 [IQR 4.89, 5.76] in non-survivors compared to survivors who had a median value of 9.52 [IQR 9.52,9.52]. Supplementary File 5a shows the temporal data according to race and Supplementary File 5b shows the temporal data according to death.

**Fig. 2:**
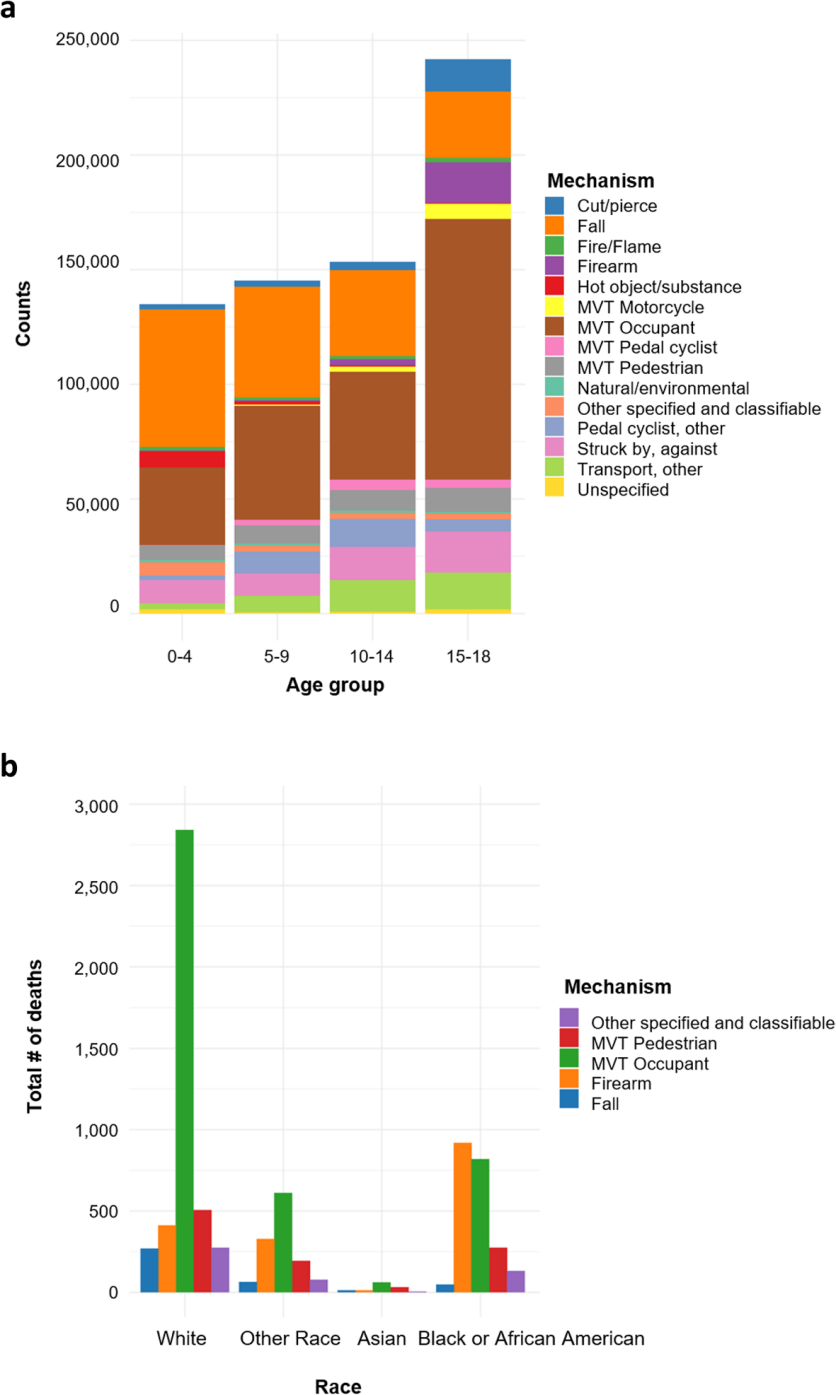
**a** Mechanisms of Mortality by Age. **b** Mechanisms of Mortality by Race

Figure 2a shows the top mechanisms of death by age, with motor vehicle collisions being the top cause of death in all ages followed by falls, penetrative injuries, and firearms respectively. Examining mortality by race (Supplemental File 3a), Black patients admitted to the hospital were significantly more likely to die (2.0% mortality rate). White patients on the other hand, were not only our largest cohort, but were significantly more likely to survive than not with a 1.2% mortality rate.

As can be seen in Fig. 2b, Black children not only had a substantially higher percentage of deaths from firearms than any other race, but firearms were the top cause of mortality in this group, whereas death as a motor vehicle occupant was the top cause of death in every other race. Mortality due to firearm injuries was the lowest in Asian children compared to other groups, as illustrated by the top 5 mechanisms depicted in the figure.

### Derivation of prediction model

In sum, 10 predictive variables (candidate predictors) were placed into the model. After backward logistic regression, eight variables remained significant: temperature, race, age, GCS, injury type, and systolic blood pressure (**c**ardiac measure) + mechanism of injury and sex. For short, we will refer to the new model as ‘TRAGIC + ’ using the first initial of each predictor. The variance inflation factor for the eight variables ranged from 1.0 to 2.5.

### AUC of model

**Fig. 3:**
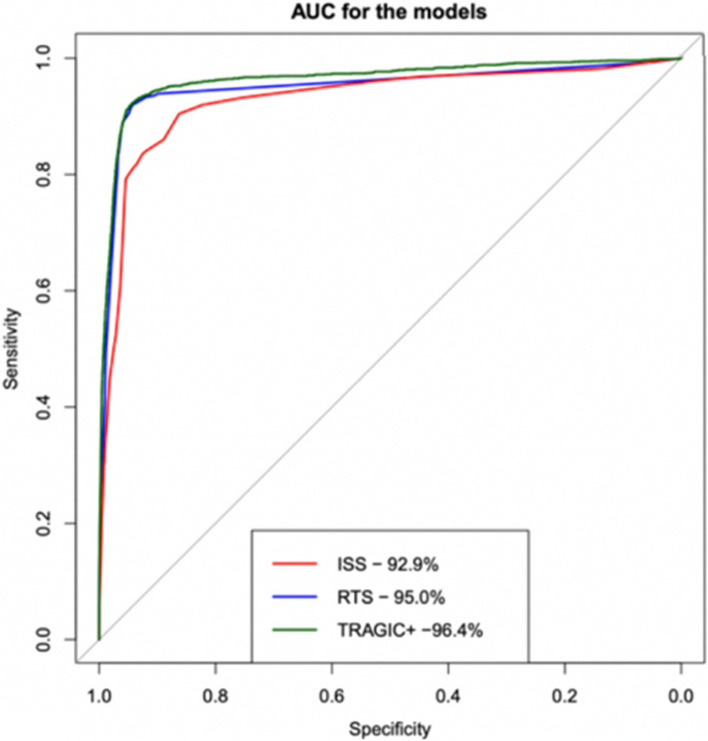
Discriminatory ability of Injury Severity Score (ISS), Revised Trauma Score (RTS), and TRAGIC + model for pediatric mortality

**Fig. 4:**
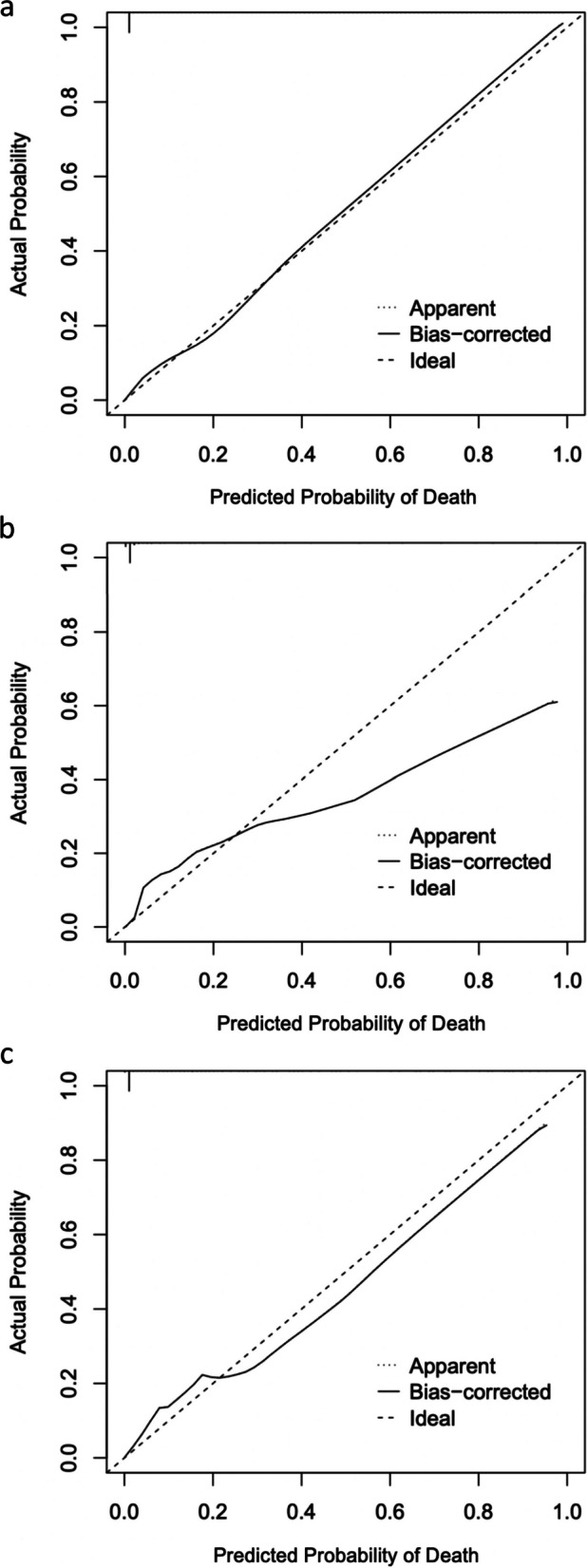
**a** TRAGIC + Calibration Plot. **b** Injury Severity Score (ISS) Calibration Plot. **c** Revised Trauma Score (RTS) Calibration Plot

Randomly allocating 80% (*n* = 554,554) of pediatric NTDB patients with a trauma-related injury were used to create the TRAGIC + prediction model, while the remaining 20% (*n* = 138,638) were used for internal validation (Fig. 1). The training and test cohorts were similar (Table 1). The TRAGIC + model yielded an AUC of 96.4% (95% CI: 95.9%-96.9%) with a sensitivity and specificity score of 92.2% and 94.4%, respectively (see Fig. 3 and Table 2). All of the AUCs from Fig. 3 are from internal validation. Calibration plots for the temporal validation as well as a tabulated summary of the Brier’s scores can be viewed in Supplementary File 6. We compared our prediction model to the ISS and RTS. The ISS yielded an AUC of 92.9% (95% CI: 92.2%-93.6%) while the RTS resulted in an AUC of 95.0% (95% CI: 94.4%-95.6%). DeLong’s test demonstrated differences between the TRAGIC + and ISS (*p* < 0.01),as well as the TRAGIC + and RTS (*p* = 0.004). Other performance metrics can be seen in Table 2. Figure 4a, b, and c display the calibration plots of all three models for comparison and shows that the TRAGIC + model is closest to the ideal. Detailed model discriminatory abilities can be viewed in Supplementary File 7.

**Table 2:** Detailed Metrics of ISS, RTS, and TRAGIC + Models

*Model*	*AUC*	*Sensitivity*	*Specificity*	*NPV*	*PPV*	*Accuracy*	*AIC*	*Youden’s (sens, spec)*	*Brier Score*
*ISS*	*92.9% (92.2%-93.6%)*	*51.1%*	*98.7%*	*99.9%*	*8.1%*	*98.6%*	*54,093*	*86.2%, 90.4%*	*0.012*
*RTS*	*95.0% (94.4%-95.6%)*	*78.8%*	*98.9%*	*99.9%*	*15.2%*	*98.7%*	*40,392*	*94.3%, 91.9%*	*0.010*
*TRAGIC* +	*96.4% (95.9%-96.9%)*	*76.1%*	*98.8%*	*99.2%*	*16.7%*	*98.8%*	*37,318*	*94.4%, 92.2%*	*0.009*

### Misclassified patients

Despite the high specificity and NPV of the TRAGIC + model, there were inevitably some instances in which the model did not accurately predict mortality. This occurred in 1,732 patients, which was approximately 1% of all patients included in the test data. The majority of these subjects were male (68%), White (61%), and had a blunt injury (78%). Overall this cohort is highly representative of the study population as a whole including patients who were not misclassified. Other characteristics of this cohort can be seen in Supplementary File 4.

### Dynamic nomogram

**Fig. 5:**
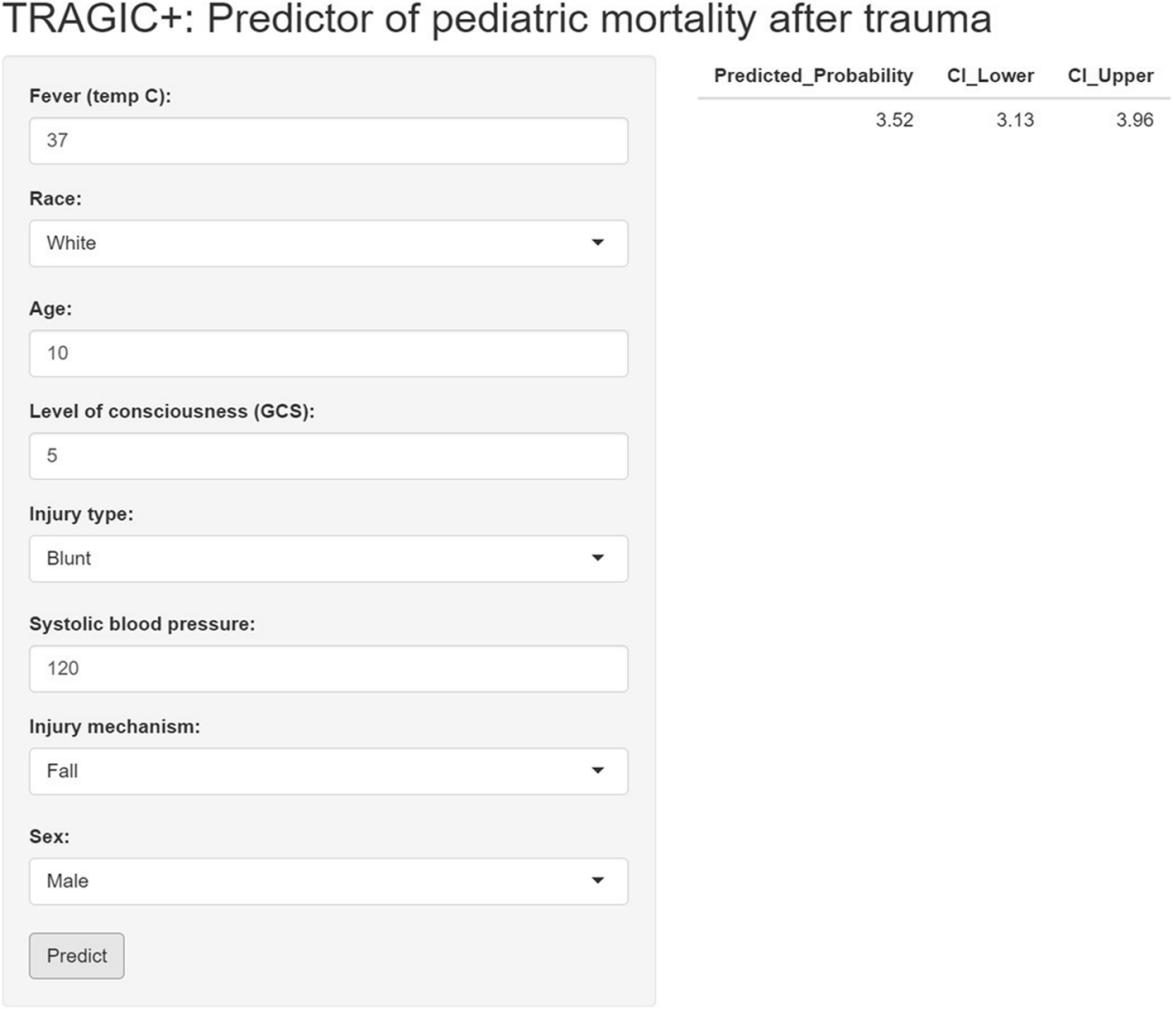
Dynamic Nomogram of TRAGIC +

The TRAGIC + prediction model was also used to develop a web-based application. In this dynamic nomogram providers can use a series of drop-down menus or a radio button to predict the probability of mortality with a 95% confidence interval. The numerical summary tabulates the predictions and the model summary tab summarizes the model with odds ratios and 95% confidence intervals for transparency. For example, an Asian 9-year-old female who received a blunt trauma after a motor vehicle trauma in which she was a pedestrian who presents with a temperature of 37 degree Celsius, a systolic blood pressure of 8, and a GCS of 7 has a mortality probability of 10.8% with a 95% confidence interval between 8.7% and 13.2%. The dynamic nomogram can be found at: https://agmoreir.shinyapps.io/TRAGIC/ (see Fig. 5). The web application provides the predicted probability for mortality with ranges of the 95% confidence interval. See Table 3 for logistic regression output values.

**Table 3:** Predictors and coefficients of the TRAGIC + model

Variable	Beta Coefficient	95% Confidence Interval	*p*-value
**Intercept**	2.866	2.578, 3.155	< 0.01
Glasgow coma scale	-0.453	-0.460, -0.446	< 0.01
Systolic blood pressure	-0.020	-0.021, -0.020	< 0.01
Temperature	-0.035	-0.042, -0.029	< 0.01
Sex	0.086	0.043, 0.129	< 0.01
***Mechanism***			
Fall	-0.507	-0.686, -0.327	< 0.01
Cut/Pierce	10.202	-737.676, 758.081	0.97
Fire/Flame	1.446	0.692, 2.201	< 0.01
Firearm	12.045	-735.833, 759.924	0.98
Hot Object/Substance	-	-	-
Machinery	-0.390	-1.267, 0.488	0.38
MVT Unspecified	-0.454	-2.782, 1.874	<0.01
MVT Motorcyclist	0.485	0.245, 0.725	< 0.01
MVT Occupant	0.618	0.479, 0.757	< 0.01
MVT Pedal Cyclist	0.646	0.426, 0.867	< 0.01
MVT Pedestrian	0.837	0.677, 0.997	< 0.01
MVT Other	0.636	0.282, 0.989	< 0.01
Natural/Environmental Bites and Stings	-1.724	-4.292, 0.844	-<0.01
Natural/environmental, other	-1.773	-4.143, 0.597	<0.01
Overexertion	-11.466	-131.160, 108.228	0.84
Pedal cyclist, other	-0.915	-1.286, -0.543	< 0.01
Pedestrian, other	-0.757	0.412, 1.103	< 0.01
Struck by, against	0.096	-0.103, 0.295	0.34
Poisoning	-1.313	-3.821, 1.196	-<0.01
Suffocation	0.753	-1.600, 3.106	0.04
Drowning/submersion	0.204	-2.131, 2.538	0.68
Adverse effects, medical care	-13.212	-517.522, 491.098	0.96
Other specified	0.275	-2.043, 2.593	>0.05
***Race***			
White	-	-	-
Asian	0.238	0.018, 0.457	< 0.01
Black or African American	0.314	0.241, 0.386	< 0.01
Other	0.103	0.025, 0.182	< 0.01
***Age***	0.030	0.025, 0.035	< 0.01
***Injury type***			
Blunt	-	-	-
Burn	-0.880	-1.605, -0.155	< 0.05
Penetrating	-9.853	-757.732, 738.025	-
Other/Unspecified	1.242	-1.076, 3.560	-

*MVT* Motor Vehicle Trauma, *RR* Respiratory rate, *SBP* Systolic blood pressure

## Discussion

Pediatric trauma is the number one leading cause of mortality in children in the United States and most deaths occur within 24 h of injury [20]. Therefore, in this study we opted to accurately predict mortality after hospital admission in pediatric patients suffering a traumatic injury. Age, GCS, systolic blood pressure, mechanism of injury, injury type, sex, temperature, and race were found to be the best predictors of mortality and were significant enough to generate a prediction-based web application. This study highlighted several important trends in mortality rates for pediatric trauma patients. Some of the most notable trends include: black patients faced significantly higher mortality rates than their white counterparts, raising the question of what role race plays in pediatric mortality, and firearm and motor vehicle injuries accounted for most of the traumatic injuries analyzed in this study. These trends are discussed in further detail below. The model used in this study, TRAGIC + , demonstrated higher discrimination, calibration, specificity, and sensitivity when compared to already established models such as the ISS and RTS.

Significant sociodemographic discrepancies among different races were observed in this analysis. Specifically, Black patients admitted to the hospital had a significantly higher mortality rate than that of White patients. Previous studies have also demonstrated the remarkable difference between the mortality outcomes of different races. For example, Haider et al. found not only that minority patients experience less favorable outcomes after a traumatic injury than their White counterparts, but that they also tend to live near trauma centers that have overall worse than expected overall mortality rates, which they argue may explain the racial disparities in trauma related outcomes. This was conducted by analyzing over 500,000 patients with an Injury Severity Score greater than or equal to 9 at Level I and II trauma centers in the National Trauma Bank between 2007–2010 [21]. Another study also concluded that relative to White patients, Black and Asian patients had a higher risk of death after injury using data from the Healthcare Cost and Utilization Project from 1998 to 2002 while controlling for various variables such as race and gender [22].

Firearm injuries and motor vehicle collisions have been among the top causes of traumatic injuries for years and as expected, this trend was also detected in our study. Other studies have also observed this pattern including McGough et al. who use data from CDC Wonder 2020 Underlying Cause of Death database and the IHME Global Burden of Disease 2019 study. They not only found that firearms and motor vehicle injuries were the number one and two causes of death in children in the United States respectively, but they also concluded that no other similarly wealthy or large country in the world has firearm deaths even in their top four causes of mortality, let alone the number one cause of death in children [23]. In additional findings from the Global Burden of Disease and Injuries study, it was found that motor vehicle accidents were responsible for 1.3 million deaths in 2010, making it the leading cause of death. This was about 50% more than what it was two decades earlier [24]. Most studies have also concluded that firearm and motor vehicle injuries are the most common causes of trauma- related mortality, but those that have looked at too broad or too specific of a population. For instance, Krug et al. conducted an epidemiologic analysis of emergency- based surveillance data for 990 infants less than 12 months old from 1994 to 2000 and found that falls were the leading cause of injury [25]. Due to the relatively small sample size and specific population, this is not generalizable to all children.

Age, GCS, systolic blood pressure, sex, intent of injury, race and mechanism of injury were found to be the best determinants of mortality in our prediction model tool. Many published and established studies have also incorporated these variables into their prediction models. The Prehospital Injury Mortality Score (PIMS) developed a tool to predict blunt trauma mortality using only prehospital variables such as age, mechanism of injury, sex, and trauma activation criterion. Their validation and derivation groups each consisted of over 160,000 patients and they displayed good discrimination with AUC of 0.79 in both groups [26]. Driessen et al. also developed a mortality prediction model in all age groups after trauma using variables such as systolic blood pressure, GCS, age, best motor response (BMR), etc. using over 300,000 patients. Therefore, this model and the PIMS model are both well calibrated and demonstrated good discrimination using variables similar to that of our prediction model including age, GCS, mechanism of injury, and systolic blood pressure [27].

Finally, race proved to be a significant determinant of mortality among pediatric trauma patients and was used in the TRAGIC + prediction model. This is seen in many other studies, one such example being a study conducted by Haider et al. in which a systematic review and meta-analysis including thirty-five studies demonstrated that black race is associated with a higher odds of death in trauma when compared with white race [28].

We found that firearm injuries and motor vehicle collisions were the leading causes of traumatic injuries, consistent with findings from other studies. Our prediction model incorporated variables that have been widely established in previous models, such as age, GCS, systolic blood pressure, sex, intent of injury, race, and mechanism of injury. The TRAGIC + model demonstrated higher discrimination, calibration, specificity, and sensitivity compared to established models like the ISS and RTS. By outperforming these widely used models, our prediction model presents a significant advancement in risk stratification and prognostication for pediatric trauma patients. Furthermore, the development of a live web application for the model enhances its accessibility and usability for healthcare providers, facilitating its potential adoption in clinical practice. Overall, the unique combination of easily accessible variables, superior predictive performance, and user-friendly implementation distinguishes our prediction model as a potentially valuable tool in improving outcomes for pediatric trauma patients.

Strengths of our project include that the TRAGIC + prediction model was created using a large sample size from a large national data bank, while adhering to all 22 of the TRIPOD guidelines (Supplemental File 1). This model was also successfully internally validated**.** Temporal validation is also a strength of the study, the participants of this cohort were not part of the derivation cohort, meaning the temporal validation is an external validation. As was seen in the results, comparing the TRAGIC + prediction model to the ISS and the RTS demonstrated that our model had higher discrimination, calibration, specificity, and sensitivity than even these published and well-known models. The derived model exhibited reasonable calibration, while both the ISS model and the RTS model display varying degrees of underfitting and overfitting, which renders their estimations unstable and less suitable for application in the NTDB. In addition to being statistically superior to these widely used models, we have also created a live web application in order to make our TRAGIC + model an easily usable and accessible tool that will further encourage healthcare providers to adopt this model.

Despite the fact that our model has a lot of potential, limitations to our study were inevitable. We were unable to validate PMIS and BMR because of the low granularity of the NTDB data (e.g., predictors not available). The TRAGIC + model also needs to be validated in other populations to increase its generalizability. TRAGIC + is also a retrospective, rather than prospective study, which can be seen both as an advantage and disadvantage. Clinicians should use their best judgment to make the decisions that are in the best interest of the patient and provide the best treatments possible.

Overall, we were able to develop a prediction model for pediatric mortality following a traumatic injury utilizing easily assessable and universally applicable clinical variables. This prediction model not only proved to be more accurate than already established models, but it was also developed into a user-friendly web application for clinical use: https://agmoreir.shinyapps.io/TRAGIC/. Future research includes validation of the model in more current NTDB databases and external validation. Conducting prospective studies to validate the prediction model in real-time clinical settings would be the ultimate goal as this can help determine the feasibility and clinical utility of the model and potential implementation process.

### Supplementary Information

**Additional file 1.** TRIPOD Checklist: Prediction Model Development and Validation.

** Additional file 2:** **Supplementary file 2a.** Imputed Study Characteristics by Death. **Supplementary file 2b.** Non-Imputed Study Characteristics by Death.

** Additional file 3:** **Supplementary file 3a.** Imputed Study Characteristics by Race. **Supplementary file 3a.** Non-Imputed Study Characteristics by Race.

** Additional file 4:** **Supplementary Figure 4a.** Classification of Variables as Factor, Integer (Int), or Numerical (Num). **Supplementary Figure 4b.** Missing Data Profile.

** Additional file 5:**  **Supplementary file 5a.** Temporal Study Characteristics by Race.  **Supplementary file 5b.** Temporal Study Characteristics by Death

** Additional file 6.** Temporal Calibration Plots and Brier’s Scores for TRAGIC, RTS, and ISS Models.

** Additional file 7.** 

## Data Availability

The datasets generated and/or analyzed during the current study are available in the American College of Surgeons’ National Trauma Data Bank repository, https://www.facs.org/quality-programs/trauma/quality/national-trauma-data-bank/reports-and-publications/.

## Abbreviations

AUC: Area under the receiver operating characteristic curve
BMR: Best Motor Response
CDC: U.S. Centers for Disease Control and Prevention
ED: Emergency Department
GCS: Glasgow Coma Scale
IHME: Institute for Health Metrics and Evaluation
INR: International Normalized Ratio
ISS: Injury Severity Score
MVT: Motor Vehicle Trauma
NTDB: National Trauma Data Bank
PIMS: Prehospital Injury Mortality Score
ROC: Receiver operating characteristic curve
RTS: Revised Trauma Score
TRAGIC +: Name of the prediction model, anagram for the variables considered: Mechanism of injury, Age, GCS, Intent of Injury, Cardiac-systolic blood pressure
TRIPOD: Transparent Reporting of a Multivariable Prediction Model for Individual Prognosis or Diagnosis
