# Supplementary material for: Development and validation of a pediatric model predicting trauma-related mortality
Source: BMC Pediatr. 2023 Dec 18;23:637. doi: 10.1186/s12887-023-04437-9 (PMC10726606; doi:10.1186/s12887-023-04437-9)
Supplement: Supplementary file 2 — Additional file 2: Supplementary file 2a. Imputed Study Characteristics by Death. Supplementary file 2b. Non-Imputed Study Characteristics by Death. [file 12887_2023_4437_MOESM2_ESM.zip › Supplementary File 2a.docx]

Imputed Study Characteristics by Death

| ***Variable*** | **Overall**, N = 779,097 | **Survived**, N = 768,263 | **Died**, N = 10,834 | **p-value** |
| --- | --- | --- | --- | --- |
| **Injury Severity Score** | 5 (4, 10) | 5 (4, 9) | 29 (25, 38) | <0.001 |
| **Glasgow Coma Score** | 15.00 (15.00, 15.00) | 15.00 (15.00, 15.00) | 3.00 (3.00, 3.00) | <0.001 |
| **Systolic Blood Pressure** | 123 (111, 135) | 123 (112, 135) | 108 (79, 134) | <0.001 |
| **Pulse** | 100 (85, 116) | 100 (85, 116) | 107 (75, 135) | <0.001 |
| **Respiratory Rate** | 20.0 (18.0, 24.0) | 20.0 (18.0, 24.0) | 16.0 (0.0, 21.0) | <0.001 |
| **Temperature** | 36.80 (36.30, 37.00) | 36.80 (36.30, 37.00) | 36.00 (35.00, 36.70) | <0.001 |
| **Gender** |  |  |  | <0.001 |
| Male | 521,334 (67%) | 513,743 (67%) | 7,591 (70%) |  |
| Female | 257,763 (33%) | 254,520 (33%) | 3,243 (30%) |  |
| **Race** |  |  |  | <0.001 |
| White | 510,604 (66%) | 504,485 (66%) | 6,119 (56%) |  |
| Other Race | 115,621 (15%) | 113,894 (15%) | 1,727 (16%) |  |
| Asian | 13,896 (1.8%) | 13,724 (1.8%) | 172 (1.6%) |  |
| Black or African American | 138,976 (18%) | 136,160 (18%) | 2,816 (26%) |  |
| **Injury Type** |  |  |  | <0.001 |
| Blunt | 680,357 (87%) | 672,512 (88%) | 7,845 (72%) |  |
| Burn | 16,453 (2.1%) | 16,319 (2.1%) | 134 (1.2%) |  |
| Other/unspecified | 33,841 (4.3%) | 32,955 (4.3%) | 886 (8.2%) |  |
| Penetrating | 48,446 (6.2%) | 46,477 (6.0%) | 1,969 (18%) |  |
| **Intent of Injury** |  |  |  | <0.001 |
| Assault | 44,499 (5.7%) | 42,383 (5.5%) | 2,116 (20%) |  |
| Other | 672 (<0.1%) | 636 (<0.1%) | 36 (0.3%) |  |
| Self-inflicted | 4,844 (0.6%) | 4,499 (0.6%) | 345 (3.2%) |  |
| Undetermined | 3,243 (0.4%) | 3,101 (0.4%) | 142 (1.3%) |  |
| Unintentional | 725,839 (93%) | 717,644 (93%) | 8,195 (76%) |  |
| **Mechanism of Injury** |  |  |  |  |
| Adverse effects, drugs | 62 (<0.1%) | 61 (<0.1%) | 1 (<0.1%) |  |
| Adverse effects, medical care | 82 (<0.1%) | 82 (<0.1%) | 0 (0%) |  |
| Cut/pierce | 25,078 (3.2%) | 24,895 (3.2%) | 183 (1.7%) |  |
| Drowning/submersion | 666 (<0.1%) | 565 (<0.1%) | 101 (0.9%) |  |
| Fall | 196,858 (25%) | 196,419 (26%) | 439 (4.1%) |  |
| Fire/flame | 6,227 (0.8%) | 6,104 (0.8%) | 123 (1.1%) |  |
| Firearm | 23,324 (3.0%) | 21,540 (2.8%) | 1,784 (16%) |  |
| Hot object/substance | 10,226 (1.3%) | 10,215 (1.3%) | 11 (0.1%) |  |
| Machinery | 2,611 (0.3%) | 2,602 (0.3%) | 9 (<0.1%) |  |
| MVT Motorcyclist | 10,619 (1.4%) | 10,427 (1.4%) | 192 (1.8%) |  |
| MVT Occupant | 279,318 (36%) | 274,446 (36%) | 4,872 (45%) |  |
| MVT Other | 2,461 (0.3%) | 2,395 (0.3%) | 66 (0.6%) |  |
| MVT Pedal cyclist | 12,027 (1.5%) | 11,782 (1.5%) | 245 (2.3%) |  |
| MVT Pedestrian | 37,150 (4.8%) | 36,029 (4.7%) | 1,121 (10%) |  |
| MVT Unspecified | 1,659 (0.2%) | 1,559 (0.2%) | 100 (0.9%) |  |
| Natural/environmental, Bites and stings | 2,558 (0.3%) | 2,553 (0.3%) | 5 (<0.1%) |  |
| Natural/environmental, Other | 3,713 (0.5%) | 3,688 (0.5%) | 25 (0.2%) |  |
| Other specified and classifiable | 15,686 (2.0%) | 15,154 (2.0%) | 532 (4.9%) |  |
| Other specified, not elsewhere classifiable | 3,580 (0.5%) | 3,539 (0.5%) | 41 (0.4%) |  |
| Overexertion | 1,649 (0.2%) | 1,649 (0.2%) | 0 (0%) |  |
| Pedal cyclist, other | 32,155 (4.1%) | 32,105 (4.2%) | 50 (0.5%) |  |
| Pedestrian, other | 3,617 (0.5%) | 3,538 (0.5%) | 79 (0.7%) |  |
| Poisoning | 586 (<0.1%) | 581 (<0.1%) | 5 (<0.1%) |  |
| Struck by, against | 57,602 (7.4%) | 57,301 (7.5%) | 301 (2.8%) |  |
| Suffocation | 310 (<0.1%) | 242 (<0.1%) | 68 (0.6%) |  |
| Transport, other | 44,280 (5.7%) | 43,909 (5.7%) | 371 (3.4%) |  |
| Unspecified | 4,993 (0.6%) | 4,883 (0.6%) | 110 (1.0%) |  |
| **Age** | 12 (6, 16) | 12 (5, 16) | 15 (8, 17) | <0.001 |
| **Hospital Disposition** |  |  |  | <0.001 |
| Survived | 768,412 (99%) | 768,263 (100%) | 149 (1.4%) |  |
| Died | 10,685 (1.4%) | 0 (0%) | 10,685 (99%) |  |
| **Year of Discharge** |  |  |  | <0.001 |
| 2007 | 73,563 (9.4%) | 71,762 (9.3%) | 1,801 (17%) |  |
| 2008 | 81,732 (10%) | 80,487 (10%) | 1,245 (11%) |  |
| 2009 | 89,260 (11%) | 88,005 (11%) | 1,255 (12%) |  |
| 2010 | 91,264 (12%) | 90,106 (12%) | 1,158 (11%) |  |
| 2011 | 91,795 (12%) | 90,623 (12%) | 1,172 (11%) |  |
| 2012 | 93,978 (12%) | 92,878 (12%) | 1,100 (10%) |  |
| 2013 | 86,537 (11%) | 85,465 (11%) | 1,072 (9.9%) |  |
| 2014 | 85,063 (11%) | 84,081 (11%) | 982 (9.1%) |  |
| 2015 | 85,905 (11%) | 84,856 (11%) | 1,049 (9.7%) |  |
| **Emergency Department Disposition** |  |  |  | <0.001 |
| Survived | 778,379 (100%) | 768,263 (100%) | 10,116 (93%) |  |
| Died | 718 (<0.1%) | 0 (0%) | 718 (6.6%) |  |
| **Revised Trauma Score** | 9.52 (9.52, 9.52) | 9.52 (9.52, 9.52) | 5.76 (4.74, 5.76) | <0.001 |
| Median (IQR); n (%) | | | | |
| Wilcoxon rank sum test; Pearson's Chi-squared test | | | | |
