# Supplementary material for: Development and validation of a pediatric model predicting trauma-related mortality
Source: BMC Pediatr. 2023 Dec 18;23:637. doi: 10.1186/s12887-023-04437-9 (PMC10726606; doi:10.1186/s12887-023-04437-9)
Supplement: Supplementary file 2 — Additional file 2: Supplementary file 2a. Imputed Study Characteristics by Death. Supplementary file 2b. Non-Imputed Study Characteristics by Death. [file 12887_2023_4437_MOESM2_ESM.zip › Supplementary File 2b.docx]

Non-Imputed Study Characteristics by Death

| ***Variable*** | **Overall**, N = 779,097 | **Survived**, N = 768,263 | **Died**, N = 10,834 | **p-value** |
| --- | --- | --- | --- | --- |
| **Injury Severity Score** | 5 (4, 10) | 5 (4, 9) | 29 (25, 38) | <0.001 |
| Unknown | 28,900 | 28,342 | 558 |  |
| **Glasgow Coma Score** | 15 (15, 15) | 15 (15, 15) | 3 (3, 3) | <0.001 |
| Unknown | 49,589 | 49,295 | 294 |  |
| **Systolic Blood Pressure** | 123 (112, 135) | 123 (112, 135) | 107 (78, 133) | <0.001 |
| Unknown | 49,193 | 48,606 | 587 |  |
| **Pulse** | 100 (85, 116) | 100 (85, 116) | 107 (74, 135) | <0.001 |
| Unknown | 15,782 | 15,352 | 430 |  |
| **Respiratory Rate** | 20 (18, 24) | 20 (18, 24) | 16 (0, 22) | <0.001 |
| Unknown | 22,150 | 20,835 | 1,315 |  |
| **Temperature** | 36.80 (36.30, 37.00) | 36.80 (36.30, 37.00) | 36.00 (34.80, 36.60) | <0.001 |
| Unknown | 77,262 | 73,480 | 3,782 |  |
| **Gender** |  |  |  | <0.001 |
| Female | 257,619 (33%) | 254,376 (33%) | 3,243 (30%) |  |
| Male | 521,081 (67%) | 513,495 (67%) | 7,586 (70%) |  |
| Unknown | 397 | 392 | 5 |  |
| **Race** |  |  |  | <0.001 |
| Other Race | 107,728 (15%) | 106,118 (15%) | 1,610 (16%) |  |
| Asian | 12,937 (1.8%) | 12,783 (1.8%) | 154 (1.5%) |  |
| Black or African American | 129,755 (18%) | 127,099 (18%) | 2,656 (26%) |  |
| White | 476,580 (66%) | 470,882 (66%) | 5,698 (56%) |  |
| Unknown | 52,097 | 51,381 | 716 |  |
| **Injury Type** |  |  |  | <0.001 |
| Blunt | 501,961 (84%) | 496,487 (84%) | 5,474 (65%) |  |
| Burn | 16,453 (2.7%) | 16,319 (2.8%) | 134 (1.6%) |  |
| Other/unspecified | 33,841 (5.6%) | 32,955 (5.6%) | 886 (10%) |  |
| Penetrating | 48,445 (8.1%) | 46,476 (7.8%) | 1,969 (23%) |  |
| Unknown | 178,397 | 176,026 | 2,371 |  |
| **Intent of Injury** |  |  |  | <0.001 |
| Assault | 44,340 (7.4%) | 42,280 (7.1%) | 2,060 (24%) |  |
| Other | 664 (0.1%) | 634 (0.1%) | 30 (0.4%) |  |
| Self-inflicted | 4,665 (0.8%) | 4,324 (0.7%) | 341 (4.0%) |  |
| Undetermined | 3,115 (0.5%) | 2,982 (0.5%) | 133 (1.6%) |  |
| Unintentional | 547,916 (91%) | 542,017 (92%) | 5,899 (70%) |  |
| Unknown | 178,397 | 176,026 | 2,371 |  |
| **Mechanism of Injury** |  |  |  |  |
| Adverse effects, drugs | 62 (<0.1%) | 61 (<0.1%) | 1 (<0.1%) |  |
| Adverse effects, medical care | 82 (<0.1%) | 82 (<0.1%) | 0 (0%) |  |
| Cut/pierce | 25,078 (4.2%) | 24,895 (4.2%) | 183 (2.2%) |  |
| Drowning/submersion | 666 (0.1%) | 565 (<0.1%) | 101 (1.2%) |  |
| Fall | 196,858 (33%) | 196,419 (33%) | 439 (5.2%) |  |
| Fire/flame | 6,227 (1.0%) | 6,104 (1.0%) | 123 (1.5%) |  |
| Firearm | 23,323 (3.9%) | 21,539 (3.6%) | 1,784 (21%) |  |
| Hot object/substance | 10,226 (1.7%) | 10,215 (1.7%) | 11 (0.1%) |  |
| Machinery | 2,611 (0.4%) | 2,602 (0.4%) | 9 (0.1%) |  |
| MVT Motorcyclist | 10,619 (1.8%) | 10,427 (1.8%) | 192 (2.3%) |  |
| MVT Occupant | 100,922 (17%) | 98,421 (17%) | 2,501 (30%) |  |
| MVT Other | 2,461 (0.4%) | 2,395 (0.4%) | 66 (0.8%) |  |
| MVT Pedal cyclist | 12,027 (2.0%) | 11,782 (2.0%) | 245 (2.9%) |  |
| MVT Pedestrian | 37,150 (6.2%) | 36,029 (6.1%) | 1,121 (13%) |  |
| MVT Unspecified | 1,659 (0.3%) | 1,559 (0.3%) | 100 (1.2%) |  |
| Natural/environmental, Bites and stings | 2,558 (0.4%) | 2,553 (0.4%) | 5 (<0.1%) |  |
| Natural/environmental, Other | 3,713 (0.6%) | 3,688 (0.6%) | 25 (0.3%) |  |
| Other specified and classifiable | 15,686 (2.6%) | 15,154 (2.6%) | 532 (6.3%) |  |
| Other specified, not elsewhere classifiable | 3,580 (0.6%) | 3,539 (0.6%) | 41 (0.5%) |  |
| Overexertion | 1,649 (0.3%) | 1,649 (0.3%) | 0 (0%) |  |
| Pedal cyclist, other | 32,155 (5.4%) | 32,105 (5.4%) | 50 (0.6%) |  |
| Pedestrian, other | 3,617 (0.6%) | 3,538 (0.6%) | 79 (0.9%) |  |
| Poisoning | 586 (<0.1%) | 581 (<0.1%) | 5 (<0.1%) |  |
| Struck by, against | 57,602 (9.6%) | 57,301 (9.7%) | 301 (3.6%) |  |
| Suffocation | 310 (<0.1%) | 242 (<0.1%) | 68 (0.8%) |  |
| Transport, other | 44,280 (7.4%) | 43,909 (7.4%) | 371 (4.4%) |  |
| Unspecified | 4,993 (0.8%) | 4,883 (0.8%) | 110 (1.3%) |  |
| Unknown | 178,397 | 176,026 | 2,371 |  |
| **Age** | 12 (6, 16) | 12 (5, 16) | 15 (8, 17) | <0.001 |
| **Year of Discharge** |  |  |  | <0.001 |
| 2007 | 73,563 (9.4%) | 71,762 (9.3%) | 1,801 (17%) |  |
| 2008 | 81,732 (10%) | 80,487 (10%) | 1,245 (11%) |  |
| 2009 | 89,260 (11%) | 88,005 (11%) | 1,255 (12%) |  |
| 2010 | 91,264 (12%) | 90,106 (12%) | 1,158 (11%) |  |
| 2011 | 91,795 (12%) | 90,623 (12%) | 1,172 (11%) |  |
| 2012 | 93,978 (12%) | 92,878 (12%) | 1,100 (10%) |  |
| 2013 | 86,537 (11%) | 85,465 (11%) | 1,072 (9.9%) |  |
| 2014 | 85,063 (11%) | 84,081 (11%) | 982 (9.1%) |  |
| 2015 | 85,905 (11%) | 84,856 (11%) | 1,049 (9.7%) |  |
| **Revised Trauma Score** | 9.52 (9.52, 9.52) | 9.52 (9.52, 9.52) | 5.76 (4.74, 5.76) | <0.001 |
| **Race** |  |  |  |  |
| Other Race | 107,728 (15%) | 106,118 (15%) | 1,610 (16%) |  |
| Asian | 12,937 (1.8%) | 12,783 (1.8%) | 154 (1.5%) |  |
| Black or African American | 129,755 (18%) | 127,099 (18%) | 2,656 (26%) |  |
| White | 476,580 (66%) | 470,882 (66%) | 5,698 (56%) |  |
| Unknown | 52,097 | 51,381 | 716 |  |
| Median (IQR); n (%) | | | | |
| Wilcoxon rank sum test; Pearson's Chi-squared test | | | | |
