# Supplementary material for: Development and validation of a pediatric model predicting trauma-related mortality
Source: BMC Pediatr. 2023 Dec 18;23:637. doi: 10.1186/s12887-023-04437-9 (PMC10726606; doi:10.1186/s12887-023-04437-9)
Supplement: Supplementary file 3 — Additional file 3: Supplementary file 3a. Imputed Study Characteristics by Race. Supplementary file 3a. Non-Imputed Study Characteristics by Race. [file 12887_2023_4437_MOESM3_ESM.zip › Supplementary File 3a.docx]

Imputed Study Characteristics by Race

| ***Variable*** | **Overall**, N = 779,097 | **White**, N = 510,604 | **Other Race**, N = 115,621 | **Asian**, N = 13,896 | **Black or African American**, N = 138,976 | **p-value** |
| --- | --- | --- | --- | --- | --- | --- |
| **Death** |  |  |  |  |  | <0.001 |
| Survived | 768,263 (99%) | 504,485 (99%) | 113,894 (99%) | 13,724 (99%) | 136,160 (98%) |  |
| Died | 10,834 (1.4%) | 6,119 (1.2%) | 1,727 (1.5%) | 172 (1.2%) | 2,816 (2.0%) |  |
| **Injury Severity Score** | 5 (4, 10) | 5 (4, 10) | 5 (4, 10) | 5 (4, 9) | 5 (4, 9) | <0.001 |
| **Glasgow Coma Score** | 15.00 (15.00, 15.00) | 15.00 (15.00, 15.00) | 15.00 (15.00, 15.00) | 15.00 (15.00, 15.00) | 15.00 (15.00, 15.00) | <0.001 |
| **Systolic Blood Pressure** | 123 (111, 135) | 122 (111, 135) | 122 (110, 134) | 120 (109, 131) | 125 (112, 138) | <0.001 |
| **Pulse** | 100 (85, 116) | 100 (85, 115) | 102 (87, 120) | 101 (87, 118) | 98 (83, 115) | <0.001 |
| **Respiratory Rate** | 20.0 (18.0, 24.0) | 20.0 (18.0, 24.0) | 20.0 (18.0, 24.0) | 20.0 (18.0, 24.0) | 20.0 (18.0, 24.0) | <0.001 |
| **Temperature** | 36.80 (36.30, 37.00) | 36.70 (36.30, 37.00) | 36.80 (36.30, 37.00) | 36.80 (36.30, 37.00) | 36.80 (36.30, 37.00) | <0.001 |
| **Gender** |  |  |  |  |  | <0.001 |
| Male | 521,334 (67%) | 335,177 (66%) | 78,292 (68%) | 8,795 (63%) | 99,070 (71%) |  |
| Female | 257,763 (33%) | 175,427 (34%) | 37,329 (32%) | 5,101 (37%) | 39,906 (29%) |  |
| **Injury Type** |  |  |  |  |  | <0.001 |
| Blunt | 680,357 (87%) | 462,646 (91%) | 98,332 (85%) | 12,628 (91%) | 106,751 (77%) |  |
| Burn | 16,453 (2.1%) | 9,480 (1.9%) | 2,588 (2.2%) | 281 (2.0%) | 4,104 (3.0%) |  |
| Other/unspecified | 33,841 (4.3%) | 21,990 (4.3%) | 4,912 (4.2%) | 442 (3.2%) | 6,497 (4.7%) |  |
| Penetrating | 48,446 (6.2%) | 16,488 (3.2%) | 9,789 (8.5%) | 545 (3.9%) | 21,624 (16%) |  |
| **Intent** |  |  |  |  |  | <0.001 |
| Assault | 44,499 (5.7%) | 11,895 (2.3%) | 9,927 (8.6%) | 512 (3.7%) | 22,165 (16%) |  |
| Other | 672 (<0.1%) | 252 (<0.1%) | 138 (0.1%) | 6 (<0.1%) | 276 (0.2%) |  |
| Self-inflicted | 4,844 (0.6%) | 3,132 (0.6%) | 789 (0.7%) | 103 (0.7%) | 820 (0.6%) |  |
| Undetermined | 3,243 (0.4%) | 1,459 (0.3%) | 539 (0.5%) | 39 (0.3%) | 1,206 (0.9%) |  |
| Unintentional | 725,839 (93%) | 493,866 (97%) | 104,228 (90%) | 13,236 (95%) | 114,509 (82%) |  |
| **Mechanism** |  |  |  |  |  |  |
| Adverse effects, drugs | 62 (<0.1%) | 32 (<0.1%) | 10 (<0.1%) | 3 (<0.1%) | 17 (<0.1%) |  |
| Adverse effects, medical care | 82 (<0.1%) | 43 (<0.1%) | 17 (<0.1%) | 0 (0%) | 22 (<0.1%) |  |
| Cut/pierce | 25,078 (3.2%) | 11,745 (2.3%) | 5,589 (4.8%) | 376 (2.7%) | 7,368 (5.3%) |  |
| Drowning/submersion | 666 (<0.1%) | 474 (<0.1%) | 82 (<0.1%) | 13 (<0.1%) | 97 (<0.1%) |  |
| Fall | 196,858 (25%) | 137,178 (27%) | 29,876 (26%) | 4,567 (33%) | 25,237 (18%) |  |
| Fire/flame | 6,227 (0.8%) | 4,578 (0.9%) | 678 (0.6%) | 52 (0.4%) | 919 (0.7%) |  |
| Firearm | 23,324 (3.0%) | 4,713 (0.9%) | 4,192 (3.6%) | 169 (1.2%) | 14,250 (10%) |  |
| Hot object/substance | 10,226 (1.3%) | 4,902 (1.0%) | 1,910 (1.7%) | 229 (1.6%) | 3,185 (2.3%) |  |
| Machinery | 2,611 (0.3%) | 2,077 (0.4%) | 346 (0.3%) | 34 (0.2%) | 154 (0.1%) |  |
| MVT Motorcyclist | 10,619 (1.4%) | 7,776 (1.5%) | 1,307 (1.1%) | 115 (0.8%) | 1,421 (1.0%) |  |
| MVT Occupant | 279,318 (36%) | 188,642 (37%) | 38,454 (33%) | 4,702 (34%) | 47,520 (34%) |  |
| MVT Other | 2,461 (0.3%) | 1,663 (0.3%) | 353 (0.3%) | 30 (0.2%) | 415 (0.3%) |  |
| MVT Pedal cyclist | 12,027 (1.5%) | 6,649 (1.3%) | 2,374 (2.1%) | 280 (2.0%) | 2,724 (2.0%) |  |
| MVT Pedestrian | 37,150 (4.8%) | 16,740 (3.3%) | 8,359 (7.2%) | 1,046 (7.5%) | 11,005 (7.9%) |  |
| MVT Unspecified | 1,659 (0.2%) | 1,088 (0.2%) | 243 (0.2%) | 19 (0.1%) | 309 (0.2%) |  |
| Natural/environmental, Bites and stings | 2,558 (0.3%) | 1,875 (0.4%) | 322 (0.3%) | 21 (0.2%) | 340 (0.2%) |  |
| Natural/environmental, Other | 3,713 (0.5%) | 3,233 (0.6%) | 313 (0.3%) | 21 (0.2%) | 146 (0.1%) |  |
| Other specified and classifiable | 15,686 (2.0%) | 10,143 (2.0%) | 2,237 (1.9%) | 209 (1.5%) | 3,097 (2.2%) |  |
| Other specified, not elsewhere classifiable | 3,580 (0.5%) | 2,011 (0.4%) | 598 (0.5%) | 58 (0.4%) | 913 (0.7%) |  |
| Overexertion | 1,649 (0.2%) | 1,054 (0.2%) | 181 (0.2%) | 24 (0.2%) | 390 (0.3%) |  |
| Pedal cyclist, other | 32,155 (4.1%) | 23,484 (4.6%) | 4,488 (3.9%) | 617 (4.4%) | 3,566 (2.6%) |  |
| Pedestrian, other | 3,617 (0.5%) | 2,203 (0.4%) | 652 (0.6%) | 66 (0.5%) | 696 (0.5%) |  |
| Poisoning | 586 (<0.1%) | 362 (<0.1%) | 89 (<0.1%) | 7 (<0.1%) | 128 (<0.1%) |  |
| Struck by, against | 57,602 (7.4%) | 37,820 (7.4%) | 7,848 (6.8%) | 830 (6.0%) | 11,104 (8.0%) |  |
| Suffocation | 310 (<0.1%) | 188 (<0.1%) | 57 (<0.1%) | 10 (<0.1%) | 55 (<0.1%) |  |
| Transport, other | 44,280 (5.7%) | 37,326 (7.3%) | 4,032 (3.5%) | 322 (2.3%) | 2,600 (1.9%) |  |
| Unspecified | 4,993 (0.6%) | 2,605 (0.5%) | 1,014 (0.9%) | 76 (0.5%) | 1,298 (0.9%) |  |
| **Age** | 12 (6, 16) | 12 (6, 16) | 10 (4, 16) | 9 (5, 15) | 13 (6, 16) | <0.001 |
| **Hospital Disposition** |  |  |  |  |  | <0.001 |
| Survived | 768,412 (99%) | 504,591 (99%) | 113,908 (99%) | 13,726 (99%) | 136,187 (98%) |  |
| Died | 10,685 (1.4%) | 6,013 (1.2%) | 1,713 (1.5%) | 170 (1.2%) | 2,789 (2.0%) |  |
| **Year of Discharge** |  |  |  |  |  | <0.001 |
| 2007 | 73,563 (9.4%) | 47,426 (9.3%) | 11,092 (9.6%) | 1,205 (8.7%) | 13,840 (10.0%) |  |
| 2008 | 81,732 (10%) | 53,070 (10%) | 13,146 (11%) | 1,264 (9.1%) | 14,252 (10%) |  |
| 2009 | 89,260 (11%) | 58,301 (11%) | 13,706 (12%) | 1,410 (10%) | 15,843 (11%) |  |
| 2010 | 91,264 (12%) | 59,792 (12%) | 13,789 (12%) | 1,503 (11%) | 16,180 (12%) |  |
| 2011 | 91,795 (12%) | 60,246 (12%) | 13,313 (12%) | 1,677 (12%) | 16,559 (12%) |  |
| 2012 | 93,978 (12%) | 61,873 (12%) | 13,748 (12%) | 1,755 (13%) | 16,602 (12%) |  |
| 2013 | 86,537 (11%) | 56,889 (11%) | 13,002 (11%) | 1,596 (11%) | 15,050 (11%) |  |
| 2014 | 85,063 (11%) | 55,636 (11%) | 12,642 (11%) | 1,780 (13%) | 15,005 (11%) |  |
| 2015 | 85,905 (11%) | 57,371 (11%) | 11,183 (9.7%) | 1,706 (12%) | 15,645 (11%) |  |
| **Emergency Department Disposition** |  |  |  |  |  | <0.001 |
| Survived | 778,379 (100%) | 510,271 (100%) | 115,514 (100%) | 13,883 (100%) | 138,711 (100%) |  |
| Died | 718 (<0.1%) | 333 (<0.1%) | 107 (<0.1%) | 13 (<0.1%) | 265 (0.2%) |  |
| **Revised Trauma Score** | 9.52 (9.52, 9.52) | 9.52 (9.52, 9.52) | 9.52 (9.52, 9.52) | 9.52 (9.52, 9.52) | 9.52 (9.52, 9.52) | <0.001 |
| n (%); Median (IQR) | | | | | | |
| Pearson's Chi-squared test; Kruskal-Wallis rank sum test | | | | | | |
