# Supplementary material for: Development and validation of a pediatric model predicting trauma-related mortality
Source: BMC Pediatr. 2023 Dec 18;23:637. doi: 10.1186/s12887-023-04437-9 (PMC10726606; doi:10.1186/s12887-023-04437-9)
Supplement: Supplementary file 3 — Additional file 3: Supplementary file 3a. Imputed Study Characteristics by Race. Supplementary file 3a. Non-Imputed Study Characteristics by Race. [file 12887_2023_4437_MOESM3_ESM.zip › Supplementary File 3b.docx]

Non-Imputed Study Characteristics by Race

| ***Variable*** | **Overall**, N = 727,000 | **Other Race**, N = 107,728 | **Asian**, N = 12,937 | **Black or African American**, N = 129,755 | **White**, N = 476,580 | **p-value** |
| --- | --- | --- | --- | --- | --- | --- |
| **Death** |  |  |  |  |  | <0.001 |
| Survived | 716,882 (99%) | 106,118 (99%) | 12,783 (99%) | 127,099 (98%) | 470,882 (99%) |  |
| Died | 10,118 (1.4%) | 1,610 (1.5%) | 154 (1.2%) | 2,656 (2.0%) | 5,698 (1.2%) |  |
| **Injury Severity Score** | 5 (4, 10) | 5 (4, 10) | 5 (4, 9) | 5 (4, 9) | 5 (4, 10) | <0.001 |
| Unknown | 27,951 | 12,297 | 855 | 4,500 | 10,299 |  |
| **Glasgow Coma Score** | 15 (15, 15) | 15 (15, 15) | 15 (15, 15) | 15 (15, 15) | 15 (15, 15) | <0.001 |
| Unknown | 45,287 | 6,521 | 1,016 | 7,489 | 30,261 |  |
| **Systolic Blood Pressure** | 123 (112, 135) | 122 (110, 134) | 120 (109, 131) | 125 (113, 138) | 123 (112, 135) | <0.001 |
| Unknown | 44,612 | 6,787 | 865 | 7,066 | 29,894 |  |
| **Pulse** | 100 (85, 116) | 102 (87, 120) | 101 (86, 118) | 98 (82, 115) | 99 (85, 115) | <0.001 |
| Unknown | 14,479 | 1,904 | 187 | 2,661 | 9,727 |  |
| **Respiratory Rate** | 20 (18, 24) | 20 (18, 24) | 20 (18, 24) | 20 (18, 24) | 20 (18, 24) | <0.001 |
| Unknown | 20,199 | 2,978 | 351 | 3,310 | 13,560 |  |
| **Temperature** | 36.80 (36.30, 37.00) | 36.80 (36.30, 37.00) | 36.80 (36.30, 37.00) | 36.80 (36.30, 37.00) | 36.80 (36.30, 37.00) | <0.001 |
| Unknown | 71,898 | 12,308 | 1,160 | 15,024 | 43,406 |  |
| **Gender** |  |  |  |  |  | <0.001 |
| Female | 240,198 (33%) | 34,710 (32%) | 4,776 (37%) | 37,215 (29%) | 163,497 (34%) |  |
| Male | 486,531 (67%) | 72,976 (68%) | 8,154 (63%) | 92,512 (71%) | 312,889 (66%) |  |
| Unknown | 271 | 42 | 7 | 28 | 194 |  |
| **Injury Type** |  |  |  |  |  | <0.001 |
| Blunt | 465,825 (83%) | 70,719 (81%) | 9,043 (89%) | 70,889 (70%) | 315,174 (88%) |  |
| Burn | 15,529 (2.8%) | 2,417 (2.8%) | 259 (2.5%) | 3,879 (3.8%) | 8,974 (2.5%) |  |
| Other/unspecified | 31,497 (5.6%) | 4,564 (5.2%) | 393 (3.9%) | 6,043 (6.0%) | 20,497 (5.7%) |  |
| Penetrating | 45,634 (8.2%) | 9,238 (11%) | 507 (5.0%) | 20,414 (20%) | 15,475 (4.3%) |  |
| Unknown | 168,515 | 20,790 | 2,735 | 28,530 | 116,460 |  |
| **Intent of Injury** |  |  |  |  |  | <0.001 |
| Assault | 41,505 (7.4%) | 9,306 (11%) | 477 (4.7%) | 20,749 (20%) | 10,973 (3.0%) |  |
| Other | 625 (0.1%) | 126 (0.1%) | 5 (<0.1%) | 254 (0.3%) | 240 (<0.1%) |  |
| Self-inflicted | 4,445 (0.8%) | 715 (0.8%) | 94 (0.9%) | 735 (0.7%) | 2,901 (0.8%) |  |
| Undetermined | 2,895 (0.5%) | 466 (0.5%) | 31 (0.3%) | 1,077 (1.1%) | 1,321 (0.4%) |  |
| Unintentional | 509,015 (91%) | 76,325 (88%) | 9,595 (94%) | 78,410 (77%) | 344,685 (96%) |  |
| Unknown | 168,515 | 20,790 | 2,735 | 28,530 | 116,460 |  |
| **Mechanism of Injury** |  |  |  |  |  |  |
| Adverse effects, drugs | 62 (<0.1%) | 10 (<0.1%) | 3 (<0.1%) | 17 (<0.1%) | 32 (<0.1%) |  |
| Adverse effects, medical care | 81 (<0.1%) | 17 (<0.1%) | 0 (0%) | 21 (<0.1%) | 43 (<0.1%) |  |
| Cut/pierce | 23,344 (4.2%) | 5,239 (6.0%) | 348 (3.4%) | 6,831 (6.7%) | 10,926 (3.0%) |  |
| Drowning/submersion | 629 (0.1%) | 75 (<0.1%) | 12 (0.1%) | 93 (<0.1%) | 449 (0.1%) |  |
| Fall | 180,871 (32%) | 27,530 (32%) | 4,269 (42%) | 23,147 (23%) | 125,925 (35%) |  |
| Fire/flame | 5,948 (1.1%) | 644 (0.7%) | 48 (0.5%) | 875 (0.9%) | 4,381 (1.2%) |  |
| Firearm | 22,250 (4.0%) | 3,992 (4.6%) | 159 (1.6%) | 13,578 (13%) | 4,521 (1.3%) |  |
| Hot object/substance | 9,581 (1.7%) | 1,773 (2.0%) | 211 (2.1%) | 3,004 (3.0%) | 4,593 (1.3%) |  |
| Machinery | 2,438 (0.4%) | 324 (0.4%) | 32 (0.3%) | 140 (0.1%) | 1,942 (0.5%) |  |
| MVT Motorcyclist | 10,098 (1.8%) | 1,229 (1.4%) | 106 (1.0%) | 1,363 (1.3%) | 7,400 (2.1%) |  |
| MVT Occupant | 94,216 (17%) | 15,229 (18%) | 1,659 (16%) | 16,131 (16%) | 61,197 (17%) |  |
| MVT Other | 2,338 (0.4%) | 326 (0.4%) | 30 (0.3%) | 395 (0.4%) | 1,587 (0.4%) |  |
| MVT Pedal cyclist | 11,278 (2.0%) | 2,252 (2.6%) | 265 (2.6%) | 2,543 (2.5%) | 6,218 (1.7%) |  |
| MVT Pedestrian | 34,488 (6.2%) | 7,767 (8.9%) | 962 (9.4%) | 10,188 (10%) | 15,571 (4.3%) |  |
| MVT Unspecified | 1,529 (0.3%) | 228 (0.3%) | 16 (0.2%) | 286 (0.3%) | 999 (0.3%) |  |
| Natural/environmental, Bites and stings | 2,386 (0.4%) | 305 (0.4%) | 13 (0.1%) | 320 (0.3%) | 1,748 (0.5%) |  |
| Natural/environmental, Other | 3,535 (0.6%) | 290 (0.3%) | 19 (0.2%) | 138 (0.1%) | 3,088 (0.9%) |  |
| Other specified and classifiable | 14,561 (2.6%) | 2,065 (2.4%) | 181 (1.8%) | 2,890 (2.9%) | 9,425 (2.6%) |  |
| Other specified, not elsewhere classifiable | 3,319 (0.6%) | 555 (0.6%) | 54 (0.5%) | 836 (0.8%) | 1,874 (0.5%) |  |
| Overexertion | 1,518 (0.3%) | 165 (0.2%) | 24 (0.2%) | 364 (0.4%) | 965 (0.3%) |  |
| Pedal cyclist, other | 29,805 (5.3%) | 4,187 (4.8%) | 568 (5.6%) | 3,288 (3.2%) | 21,762 (6.0%) |  |
| Pedestrian, other | 3,353 (0.6%) | 617 (0.7%) | 61 (0.6%) | 641 (0.6%) | 2,034 (0.6%) |  |
| Poisoning | 560 (0.1%) | 85 (<0.1%) | 6 (<0.1%) | 126 (0.1%) | 343 (<0.1%) |  |
| Struck by, against | 53,463 (9.6%) | 7,235 (8.3%) | 769 (7.5%) | 10,343 (10%) | 35,116 (9.8%) |  |
| Suffocation | 293 (<0.1%) | 55 (<0.1%) | 10 (<0.1%) | 50 (<0.1%) | 178 (<0.1%) |  |
| Transport, other | 41,948 (7.5%) | 3,795 (4.4%) | 306 (3.0%) | 2,424 (2.4%) | 35,423 (9.8%) |  |
| Unspecified | 4,593 (0.8%) | 949 (1.1%) | 71 (0.7%) | 1,193 (1.2%) | 2,380 (0.7%) |  |
| Unknown | 168,515 | 20,790 | 2,735 | 28,530 | 116,460 |  |
| **Age** | 12 (6, 16) | 10 (4, 16) | 9 (5, 15) | 13 (6, 16) | 12 (6, 16) | <0.001 |
| **Year of Discharge** |  |  |  |  |  | <0.001 |
| 2007 | 64,733 (8.9%) | 9,801 (9.1%) | 1,050 (8.1%) | 12,234 (9.4%) | 41,648 (8.7%) |  |
| 2008 | 75,546 (10%) | 12,113 (11%) | 1,133 (8.8%) | 13,134 (10%) | 49,166 (10%) |  |
| 2009 | 82,458 (11%) | 12,728 (12%) | 1,287 (9.9%) | 14,656 (11%) | 53,787 (11%) |  |
| 2010 | 84,672 (12%) | 12,744 (12%) | 1,383 (11%) | 15,015 (12%) | 55,530 (12%) |  |
| 2011 | 86,055 (12%) | 12,478 (12%) | 1,566 (12%) | 15,481 (12%) | 56,530 (12%) |  |
| 2012 | 87,782 (12%) | 12,786 (12%) | 1,634 (13%) | 15,565 (12%) | 57,797 (12%) |  |
| 2013 | 81,272 (11%) | 12,190 (11%) | 1,512 (12%) | 14,156 (11%) | 53,414 (11%) |  |
| 2014 | 81,416 (11%) | 12,116 (11%) | 1,719 (13%) | 14,401 (11%) | 53,180 (11%) |  |
| 2015 | 83,066 (11%) | 10,772 (10.0%) | 1,653 (13%) | 15,113 (12%) | 55,528 (12%) |  |
| **Revised Trauma Score** | 9.52 (9.52, 9.52) | 9.52 (9.52, 9.52) | 9.52 (9.52, 9.52) | 9.52 (9.52, 9.52) | 9.52 (9.52, 9.52) | <0.001 |
| n (%); Median (IQR) | | | | | | |
| Pearson's Chi-squared test; Kruskal-Wallis rank sum test | | | | | | |
