# Supplementary material for: Development and validation of a pediatric model predicting trauma-related mortality
Source: BMC Pediatr. 2023 Dec 18;23:637. doi: 10.1186/s12887-023-04437-9 (PMC10726606; doi:10.1186/s12887-023-04437-9)
Supplement: Supplementary file 4 — Additional file 4: Supplementary Figure 4a. Classification of Variables as Factor, Integer (Int), or Numerical (Num). Supplementary Figure 4b. Missing Data Profile. [file 12887_2023_4437_MOESM4_ESM.pdf]

Variable Classification

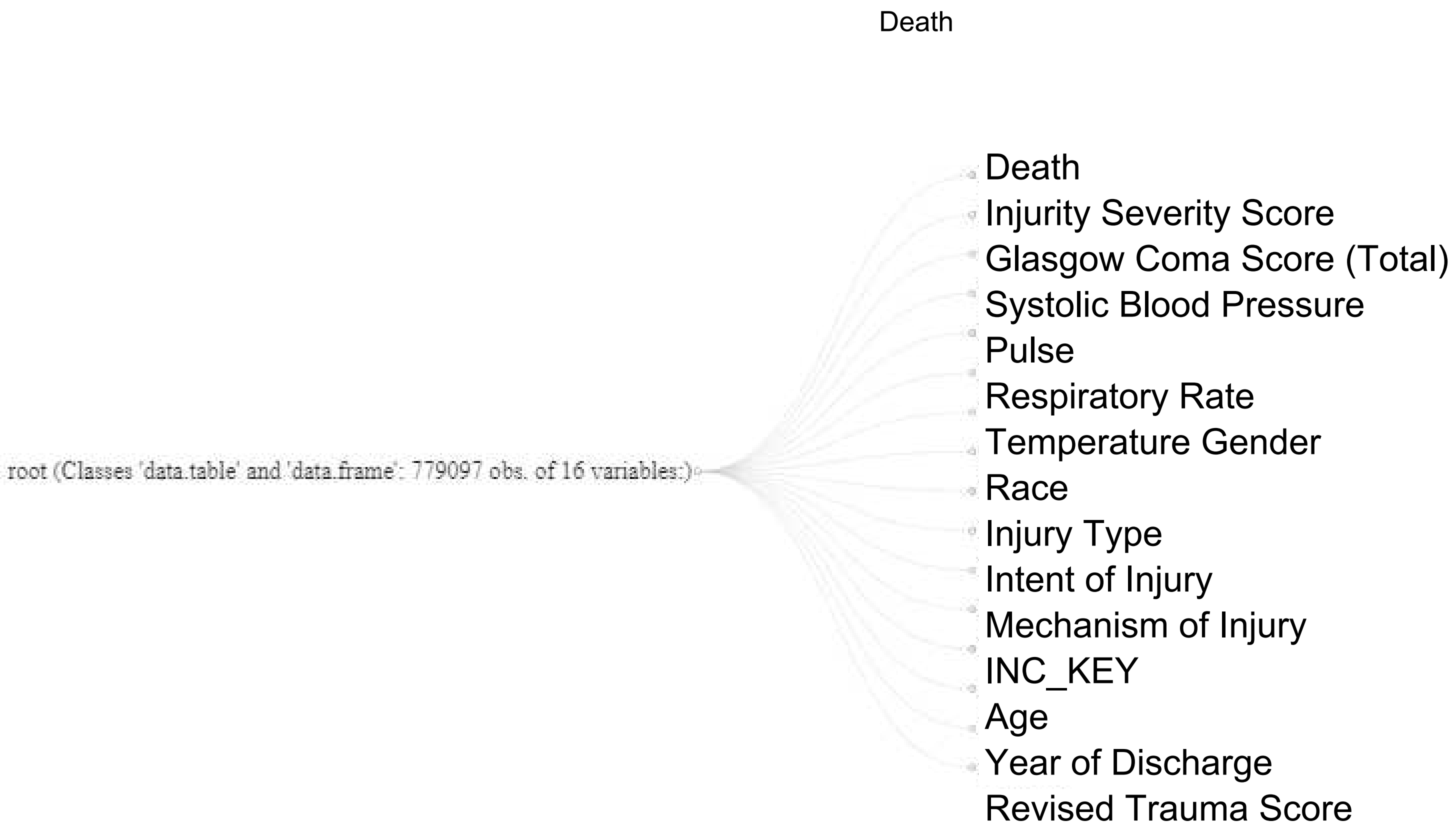

Supplementary Figure 4a. Classification of Variables as Factor, Integer (Int), or Numerical (Num)

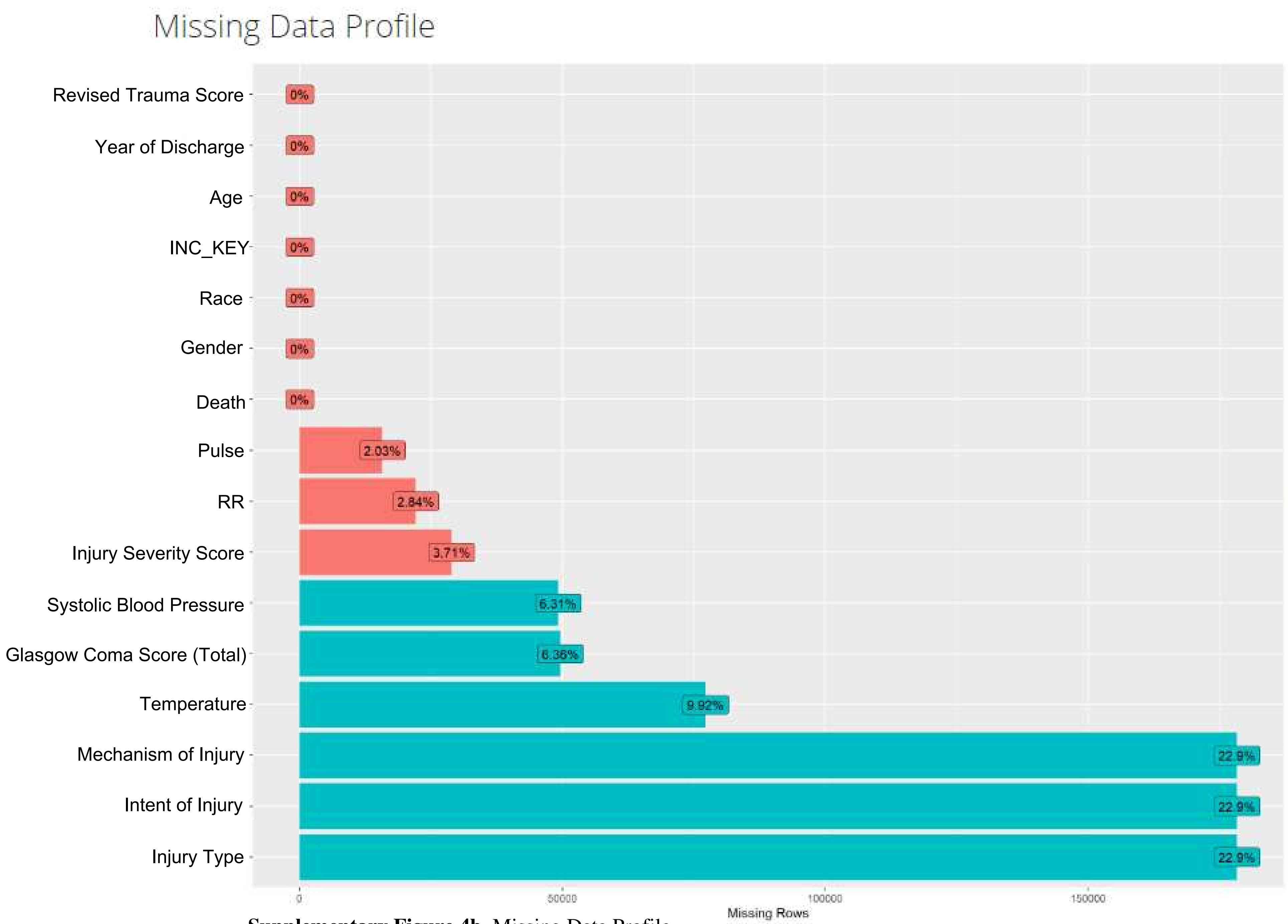

Supplementary Figure 4b. Missing Data Profile
