# Supplementary material for: Development and validation of a pediatric model predicting trauma-related mortality
Source: BMC Pediatr. 2023 Dec 18;23:637. doi: 10.1186/s12887-023-04437-9 (PMC10726606; doi:10.1186/s12887-023-04437-9)
Supplement: Supplementary file 5 — Additional file 5: Supplementary file 5a. Temporal Study Characteristics by Race. Supplementary file 5b. Temporal Study Characteristics by Death [file 12887_2023_4437_MOESM5_ESM.zip › Supplementary File 5a.docx]

Temporal Study Characteristics by Race

| ***Variable*** | **Overall**, N = 85,905 | **White**, N = 57,371 | **Other Race**, N = 11,183 | **Asian**, N = 1,706 | **Black or African American**, N = 15,645 | **p-value** |
| --- | --- | --- | --- | --- | --- | --- |
| **Death** |  |  |  |  |  | <0.001 |
| Survived | 84,856 (99%) | 56,757 (99%) | 11,040 (99%) | 1,690 (99%) | 15,369 (98%) |  |
| Died | 1,049 (1.2%) | 614 (1.1%) | 143 (1.3%) | 16 (0.9%) | 276 (1.8%) |  |
| **Injury Severity Score** | 5 (4, 9) | 5 (4, 9) | 5 (4, 9) | 5 (4, 9) | 5 (4, 9) | <0.001 |
| **Glasgow Coma Score** | 15.00 (15.00, 15.00) | 15.00 (15.00, 15.00) | 15.00 (15.00, 15.00) | 15.00 (15.00, 15.00) | 15.00 (15.00, 15.00) | <0.001 |
| **Systolic Blood Pressure** | 122 (111, 134) | 121 (111, 133) | 121 (110, 133) | 118 (109, 128) | 124 (112, 137) | <0.001 |
| **Pulse** | 100 (85, 116) | 100 (85, 116) | 102 (87, 119) | 100 (86, 116) | 98 (83, 115) | <0.001 |
| **Respiratory Rate** | 20.0 (18.0, 24.0) | 20.0 (18.0, 24.0) | 20.0 (18.0, 24.0) | 20.0 (18.0, 24.0) | 20.0 (18.0, 24.0) | <0.001 |
| **Temperature** | 36.80 (36.60, 37.10) | 36.80 (36.60, 37.10) | 36.80 (36.60, 37.10) | 36.80 (36.50, 37.10) | 36.80 (36.50, 37.10) | 0.011 |
| **Gender** |  |  |  |  |  | <0.001 |
| Female | 29,584 (34%) | 20,432 (36%) | 3,739 (33%) | 630 (37%) | 4,783 (31%) |  |
| Male | 56,321 (66%) | 36,939 (64%) | 7,444 (67%) | 1,076 (63%) | 10,862 (69%) |  |
| **Injury Type** |  |  |  |  |  | <0.001 |
| Blunt | 76,590 (89%) | 52,351 (91%) | 9,894 (88%) | 1,596 (94%) | 12,749 (81%) |  |
| Burn | 1,358 (1.6%) | 884 (1.5%) | 149 (1.3%) | 25 (1.5%) | 300 (1.9%) |  |
| Other/unspecified | 3,450 (4.0%) | 2,290 (4.0%) | 431 (3.9%) | 45 (2.6%) | 684 (4.4%) |  |
| Penetrating | 4,507 (5.2%) | 1,846 (3.2%) | 709 (6.3%) | 40 (2.3%) | 1,912 (12%) |  |
| **Intent of Injury** |  |  |  |  |  |  |
| Unintentional | 81,330 (95%) | 55,692 (97%) | 10,420 (93%) | 1,659 (97%) | 13,559 (87%) |  |
| Undetermined | 403 (0.5%) | 212 (0.4%) | 57 (0.5%) | 5 (0.3%) | 129 (0.8%) |  |
| Other | 54 (<0.1%) | 26 (<0.1%) | 7 (<0.1%) | 0 (0%) | 21 (0.1%) |  |
| Self-inflicted | 586 (0.7%) | 386 (0.7%) | 82 (0.7%) | 14 (0.8%) | 104 (0.7%) |  |
| Assault | 3,532 (4.1%) | 1,055 (1.8%) | 617 (5.5%) | 28 (1.6%) | 1,832 (12%) |  |
| **Mechanism of Injury** |  |  |  |  |  |  |
| Other specified and classifiable | 6,781 (7.9%) | 5,180 (9.0%) | 654 (5.8%) | 68 (4.0%) | 879 (5.6%) |  |
| Adverse effects, drugs | 3 (<0.1%) | 1 (<0.1%) | 1 (<0.1%) | 0 (0%) | 1 (<0.1%) |  |
| Adverse effects, medical care | 4 (<0.1%) | 2 (<0.1%) | 2 (<0.1%) | 0 (0%) | 0 (0%) |  |
| Cut/pierce | 2,483 (2.9%) | 1,351 (2.4%) | 449 (4.0%) | 32 (1.9%) | 651 (4.2%) |  |
| Drowning/submersion | 52 (<0.1%) | 41 (<0.1%) | 4 (<0.1%) | 2 (0.1%) | 5 (<0.1%) |  |
| Fall | 21,866 (25%) | 15,398 (27%) | 3,049 (27%) | 582 (34%) | 2,837 (18%) |  |
| Fire/flame | 565 (0.7%) | 428 (0.7%) | 56 (0.5%) | 6 (0.4%) | 75 (0.5%) |  |
| Firearm | 2,013 (2.3%) | 488 (0.9%) | 257 (2.3%) | 8 (0.5%) | 1,260 (8.1%) |  |
| Machinery | 276 (0.3%) | 215 (0.4%) | 42 (0.4%) | 2 (0.1%) | 17 (0.1%) |  |
| MVT Motorcyclist | 1,070 (1.2%) | 751 (1.3%) | 112 (1.0%) | 18 (1.1%) | 189 (1.2%) |  |
| MVT Occupant | 35,023 (41%) | 23,611 (41%) | 4,329 (39%) | 663 (39%) | 6,420 (41%) |  |
| MVT Other | 277 (0.3%) | 195 (0.3%) | 33 (0.3%) | 2 (0.1%) | 47 (0.3%) |  |
| MVT Pedal cyclist | 1,168 (1.4%) | 621 (1.1%) | 217 (1.9%) | 32 (1.9%) | 298 (1.9%) |  |
| MVT Pedestrian | 3,576 (4.2%) | 1,732 (3.0%) | 657 (5.9%) | 113 (6.6%) | 1,074 (6.9%) |  |
| MVT Unspecified | 197 (0.2%) | 137 (0.2%) | 18 (0.2%) | 1 (<0.1%) | 41 (0.3%) |  |
| Natural/environmental, Bites and stings | 94 (0.1%) | 72 (0.1%) | 8 (<0.1%) | 1 (<0.1%) | 13 (<0.1%) |  |
| Natural/environmental, Other | 377 (0.4%) | 320 (0.6%) | 33 (0.3%) | 4 (0.2%) | 20 (0.1%) |  |
| Other specified, not elsewhere classifiable | 414 (0.5%) | 225 (0.4%) | 61 (0.5%) | 5 (0.3%) | 123 (0.8%) |  |
| Overexertion | 96 (0.1%) | 61 (0.1%) | 8 (<0.1%) | 1 (<0.1%) | 26 (0.2%) |  |
| Pedal cyclist, other | 3,206 (3.7%) | 2,347 (4.1%) | 413 (3.7%) | 67 (3.9%) | 379 (2.4%) |  |
| Pedestrian, other | 369 (0.4%) | 237 (0.4%) | 58 (0.5%) | 4 (0.2%) | 70 (0.4%) |  |
| Poisoning | 44 (<0.1%) | 30 (<0.1%) | 8 (<0.1%) | 1 (<0.1%) | 5 (<0.1%) |  |
| Struck by, against | 5,451 (6.3%) | 3,639 (6.3%) | 635 (5.7%) | 87 (5.1%) | 1,090 (7.0%) |  |
| Suffocation | 33 (<0.1%) | 24 (<0.1%) | 3 (<0.1%) | 0 (0%) | 6 (<0.1%) |  |
| Unspecified | 467 (0.5%) | 265 (0.5%) | 76 (0.7%) | 7 (0.4%) | 119 (0.8%) |  |
| **Age** | 11 (5, 16) | 11 (6, 16) | 10 (5, 15) | 9 (5, 15) | 12 (6, 16) | <0.001 |
| **Year of Discharge** |  |  |  |  |  |  |
| 2007 | 0 (0%) | 0 (0%) | 0 (0%) | 0 (0%) | 0 (0%) |  |
| 2008 | 0 (0%) | 0 (0%) | 0 (0%) | 0 (0%) | 0 (0%) |  |
| 2009 | 0 (0%) | 0 (0%) | 0 (0%) | 0 (0%) | 0 (0%) |  |
| 2010 | 0 (0%) | 0 (0%) | 0 (0%) | 0 (0%) | 0 (0%) |  |
| 2011 | 0 (0%) | 0 (0%) | 0 (0%) | 0 (0%) | 0 (0%) |  |
| 2012 | 0 (0%) | 0 (0%) | 0 (0%) | 0 (0%) | 0 (0%) |  |
| 2013 | 0 (0%) | 0 (0%) | 0 (0%) | 0 (0%) | 0 (0%) |  |
| 2014 | 0 (0%) | 0 (0%) | 0 (0%) | 0 (0%) | 0 (0%) |  |
| 2015 | 85,905 (100%) | 57,371 (100%) | 11,183 (100%) | 1,706 (100%) | 15,645 (100%) |  |
| **Revised Trauma Score** | 9.52 (9.52, 9.52) | 9.52 (9.52, 9.52) | 9.52 (9.52, 9.52) | 9.52 (9.52, 9.52) | 9.52 (9.52, 9.52) | 0.002 |
| n (%); Median (IQR) | | | | | | |
| Pearson's Chi-squared test; Kruskal-Wallis rank sum test | | | | | | |
