# Supplementary material for: Development and validation of a pediatric model predicting trauma-related mortality
Source: BMC Pediatr. 2023 Dec 18;23:637. doi: 10.1186/s12887-023-04437-9 (PMC10726606; doi:10.1186/s12887-023-04437-9)
Supplement: Supplementary file 5 — Additional file 5: Supplementary file 5a. Temporal Study Characteristics by Race. Supplementary file 5b. Temporal Study Characteristics by Death [file 12887_2023_4437_MOESM5_ESM.zip › Supplementary File 5b.docx]

Temporal Study Characteristics by Death

| ***Variable*** | **Overall**, N = 85,905 | **Survived**, N = 84,856 | **Died**, N = 1,049 | **p-value** |
| --- | --- | --- | --- | --- |
| **Injury Severity Score** | 5 (4, 9) | 5 (4, 9) | 29 (25, 38) | <0.001 |
| **Glasgow Coma Score** | 15.00 (15.00, 15.00) | 15.00 (15.00, 15.00) | 3.00 (3.00, 3.00) | <0.001 |
| **Systolic Blood Pressure** | 122 (111, 134) | 122 (111, 134) | 108 (82, 131) | <0.001 |
| **Pulse** | 100 (85, 116) | 100 (85, 116) | 111 (80, 138) | <0.001 |
| **Respiratory Rate** | 20.0 (18.0, 24.0) | 20.0 (18.0, 24.0) | 18.0 (14.0, 24.0) | <0.001 |
| **Temperature** | 36.80 (36.60, 37.10) | 36.80 (36.60, 37.10) | 36.10 (35.20, 36.70) | <0.001 |
| **Gender** |  |  |  | <0.001 |
| Female | 29,584 (34%) | 29,280 (35%) | 304 (29%) |  |
| Male | 56,321 (66%) | 55,576 (65%) | 745 (71%) |  |
| **Race** |  |  |  | <0.001 |
| White | 57,371 (67%) | 56,757 (67%) | 614 (59%) |  |
| Other Race | 11,183 (13%) | 11,040 (13%) | 143 (14%) |  |
| Asian | 1,706 (2.0%) | 1,690 (2.0%) | 16 (1.5%) |  |
| Black or African American | 15,645 (18%) | 15,369 (18%) | 276 (26%) |  |
| **Injury Type** |  |  |  | <0.001 |
| Blunt | 76,590 (89%) | 75,767 (89%) | 823 (78%) |  |
| Burn | 1,358 (1.6%) | 1,344 (1.6%) | 14 (1.3%) |  |
| Other/unspecified | 3,450 (4.0%) | 3,368 (4.0%) | 82 (7.8%) |  |
| Penetrating | 4,507 (5.2%) | 4,377 (5.2%) | 130 (12%) |  |
| **Intent of Injury** |  |  |  |  |
| Unintentional | 81,330 (95%) | 80,482 (95%) | 848 (81%) |  |
| Undetermined | 403 (0.5%) | 384 (0.5%) | 19 (1.8%) |  |
| Other | 54 (<0.1%) | 49 (<0.1%) | 5 (0.5%) |  |
| Self-inflicted | 586 (0.7%) | 552 (0.7%) | 34 (3.2%) |  |
| Assault | 3,532 (4.1%) | 3,389 (4.0%) | 143 (14%) |  |
| **Mechanism of Injury** |  |  |  |  |
| Other specified and classifiable | 6,781 (7.9%) | 6,703 (7.9%) | 78 (7.4%) |  |
| Adverse effects, drugs | 3 (<0.1%) | 3 (<0.1%) | 0 (0%) |  |
| Adverse effects, medical care | 4 (<0.1%) | 4 (<0.1%) | 0 (0%) |  |
| Cut/pierce | 2,483 (2.9%) | 2,467 (2.9%) | 16 (1.5%) |  |
| Drowning/submersion | 52 (<0.1%) | 43 (<0.1%) | 9 (0.9%) |  |
| Fall | 21,866 (25%) | 21,816 (26%) | 50 (4.8%) |  |
| Fire/flame | 565 (0.7%) | 552 (0.7%) | 13 (1.2%) |  |
| Firearm | 2,013 (2.3%) | 1,900 (2.2%) | 113 (11%) |  |
| Machinery | 276 (0.3%) | 276 (0.3%) | 0 (0%) |  |
| MVT Motorcyclist | 1,070 (1.2%) | 1,050 (1.2%) | 20 (1.9%) |  |
| MVT Occupant | 35,023 (41%) | 34,482 (41%) | 541 (52%) |  |
| MVT Other | 277 (0.3%) | 272 (0.3%) | 5 (0.5%) |  |
| MVT Pedal cyclist | 1,168 (1.4%) | 1,147 (1.4%) | 21 (2.0%) |  |
| MVT Pedestrian | 3,576 (4.2%) | 3,459 (4.1%) | 117 (11%) |  |
| MVT Unspecified | 197 (0.2%) | 190 (0.2%) | 7 (0.7%) |  |
| Natural/environmental, Bites and stings | 94 (0.1%) | 94 (0.1%) | 0 (0%) |  |
| Natural/environmental, Other | 377 (0.4%) | 374 (0.4%) | 3 (0.3%) |  |
| Other specified, not elsewhere classifiable | 414 (0.5%) | 405 (0.5%) | 9 (0.9%) |  |
| Overexertion | 96 (0.1%) | 96 (0.1%) | 0 (0%) |  |
| Pedal cyclist, other | 3,206 (3.7%) | 3,203 (3.8%) | 3 (0.3%) |  |
| Pedestrian, other | 369 (0.4%) | 363 (0.4%) | 6 (0.6%) |  |
| Poisoning | 44 (<0.1%) | 44 (<0.1%) | 0 (0%) |  |
| Struck by, against | 5,451 (6.3%) | 5,428 (6.4%) | 23 (2.2%) |  |
| Suffocation | 33 (<0.1%) | 30 (<0.1%) | 3 (0.3%) |  |
| Unspecified | 467 (0.5%) | 455 (0.5%) | 12 (1.1%) |  |
| **Age** | 11 (5, 16) | 11 (5, 16) | 15 (7, 17) | <0.001 |
| **Year of Discharge** |  |  |  | >0.9 |
| 2007 | 0 (0%) | 0 (0%) | 0 (0%) |  |
| 2008 | 0 (0%) | 0 (0%) | 0 (0%) |  |
| 2009 | 0 (0%) | 0 (0%) | 0 (0%) |  |
| 2010 | 0 (0%) | 0 (0%) | 0 (0%) |  |
| 2011 | 0 (0%) | 0 (0%) | 0 (0%) |  |
| 2012 | 0 (0%) | 0 (0%) | 0 (0%) |  |
| 2013 | 0 (0%) | 0 (0%) | 0 (0%) |  |
| 2014 | 0 (0%) | 0 (0%) | 0 (0%) |  |
| 2015 | 85,905 (100%) | 84,856 (100%) | 1,049 (100%) |  |
| **Revised Trauma Score** | 9.52 (9.52, 9.52) | 9.52 (9.52, 9.52) | 5.76 (4.89, 5.76) | <0.001 |
| Median (IQR); n (%) | | | | |
| Wilcoxon rank sum test; Pearson's Chi-squared test; Fisher's exact test | | | | |
