# Supplementary figures and images for: Development and validation of a pediatric model predicting trauma-related mortality
Source: BMC Pediatr. 2023 Dec 18;23:637. doi: 10.1186/s12887-023-04437-9 (PMC10726606; doi:10.1186/s12887-023-04437-9)

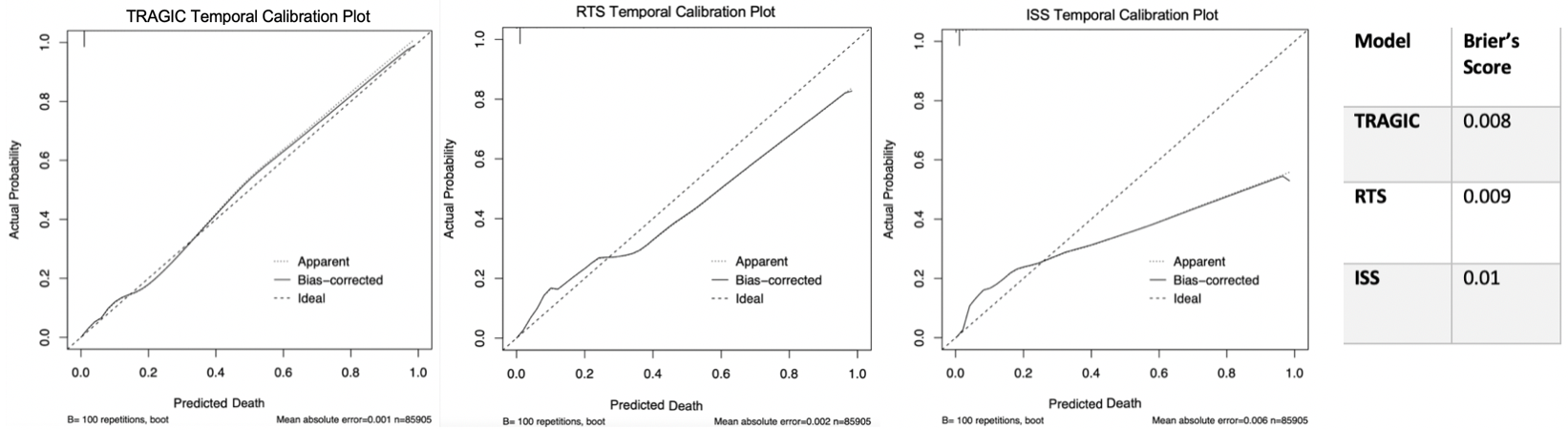


**Temporal Calibration Plots and Brier’s Scores for TRAGIC, RTS, and ISS Models**

Supplement: Supplementary file 6 — Additional file 6. Temporal Calibration Plots and Brier’s Scores for TRAGIC, RTS, and ISS Models. [file 12887_2023_4437_MOESM6_ESM.docx]
