# Supplementary material for: Development and validation of a pediatric model predicting trauma-related mortality
Source: BMC Pediatr. 2023 Dec 18;23:637. doi: 10.1186/s12887-023-04437-9 (PMC10726606; doi:10.1186/s12887-023-04437-9)
Supplement: Supplementary file 7 — Additional file 7. [file 12887_2023_4437_MOESM7_ESM.pptx]

## Slide 1
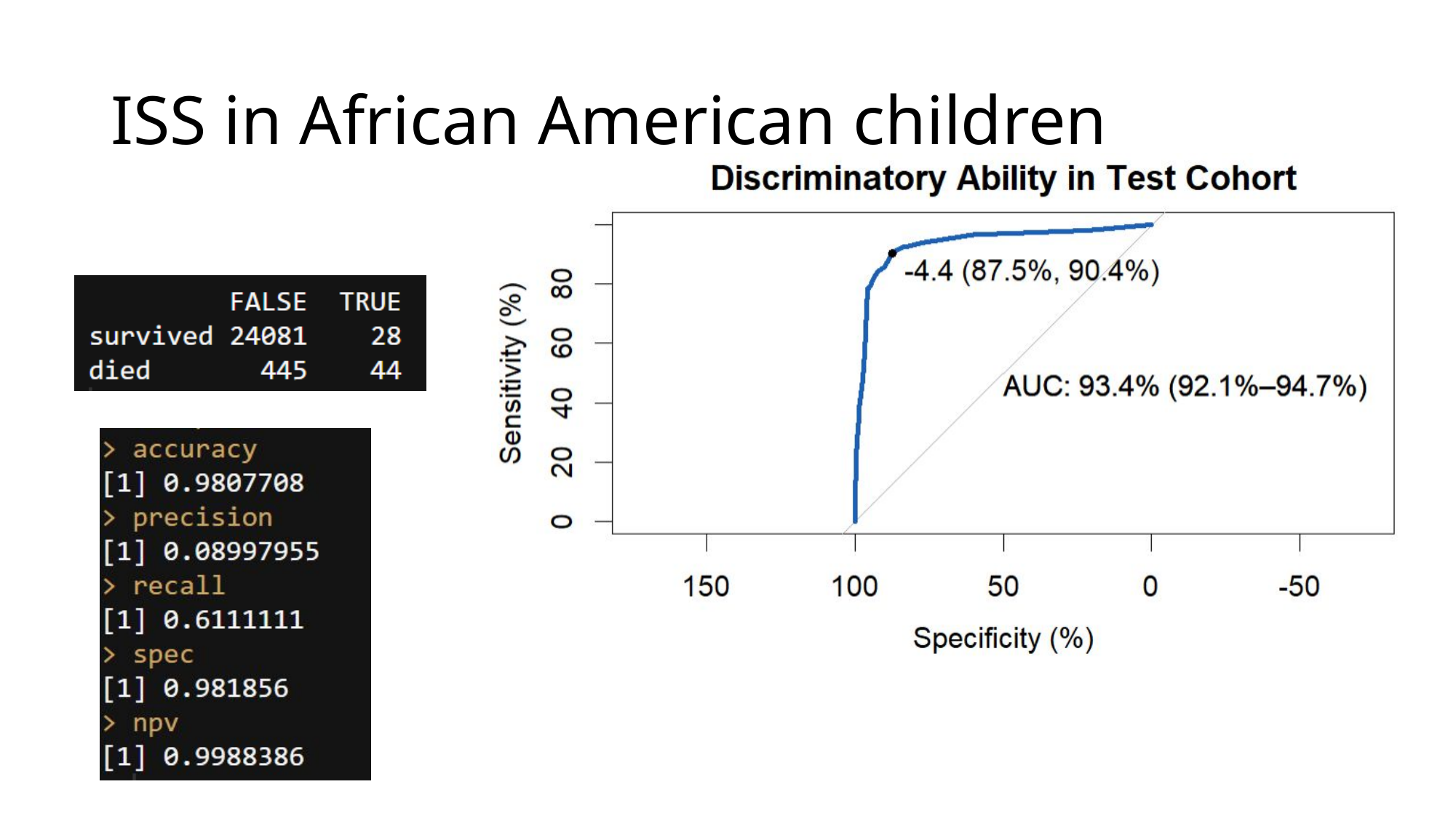

# ISS in African American children

## Slide 2
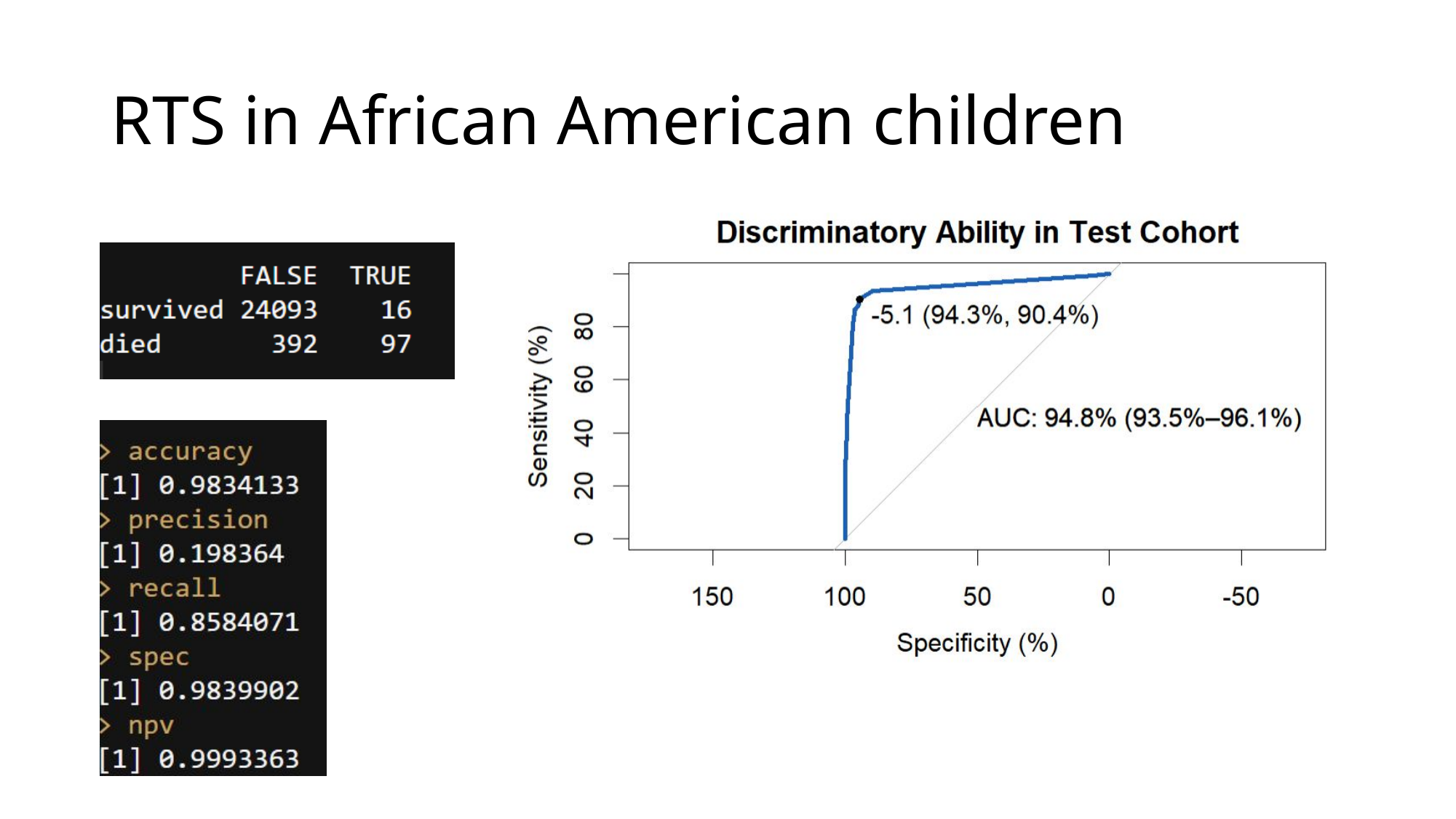

# RTS in African American children

## Slide 3
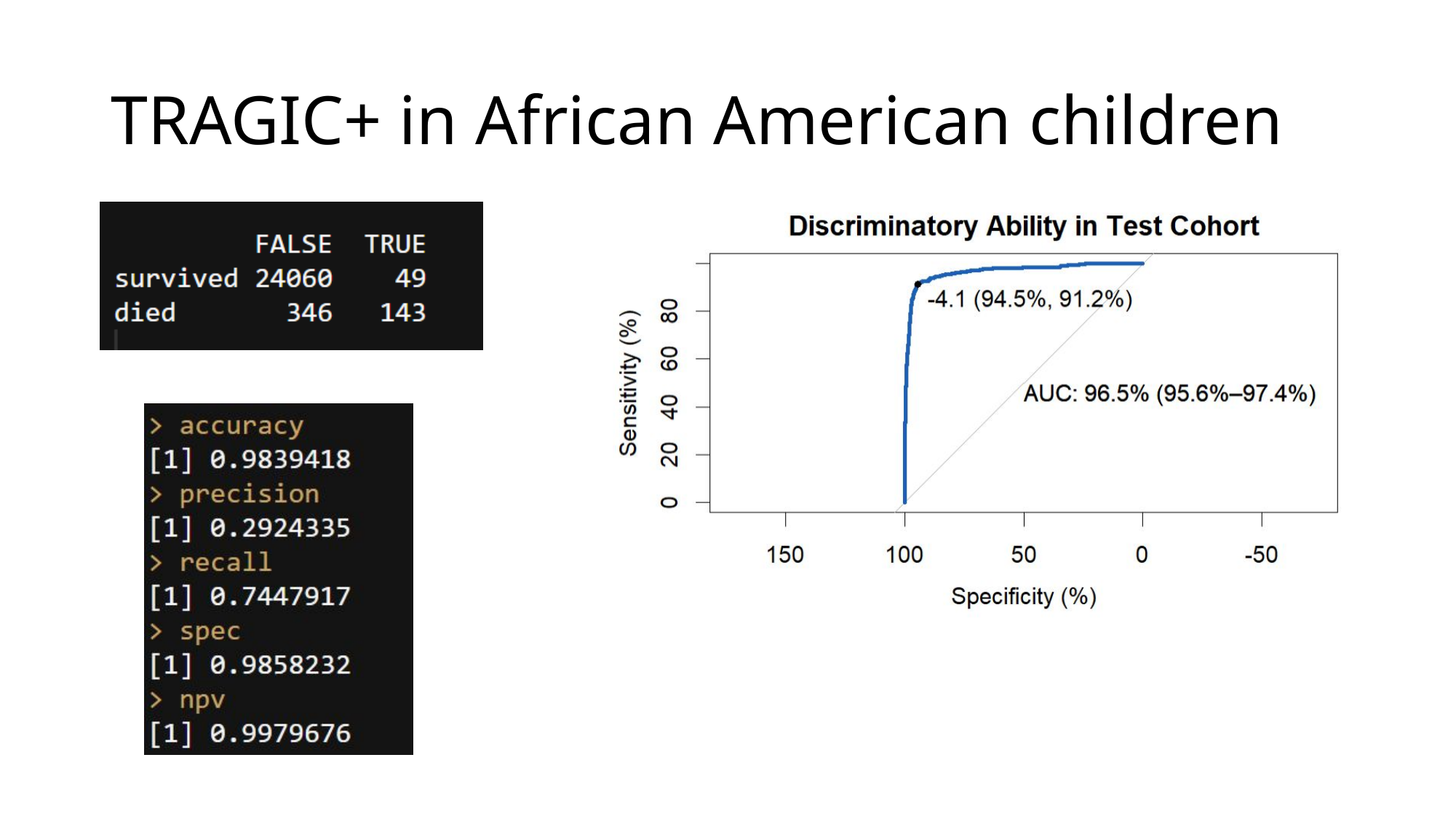

# TRAGIC+ in African American children

## Slide 4
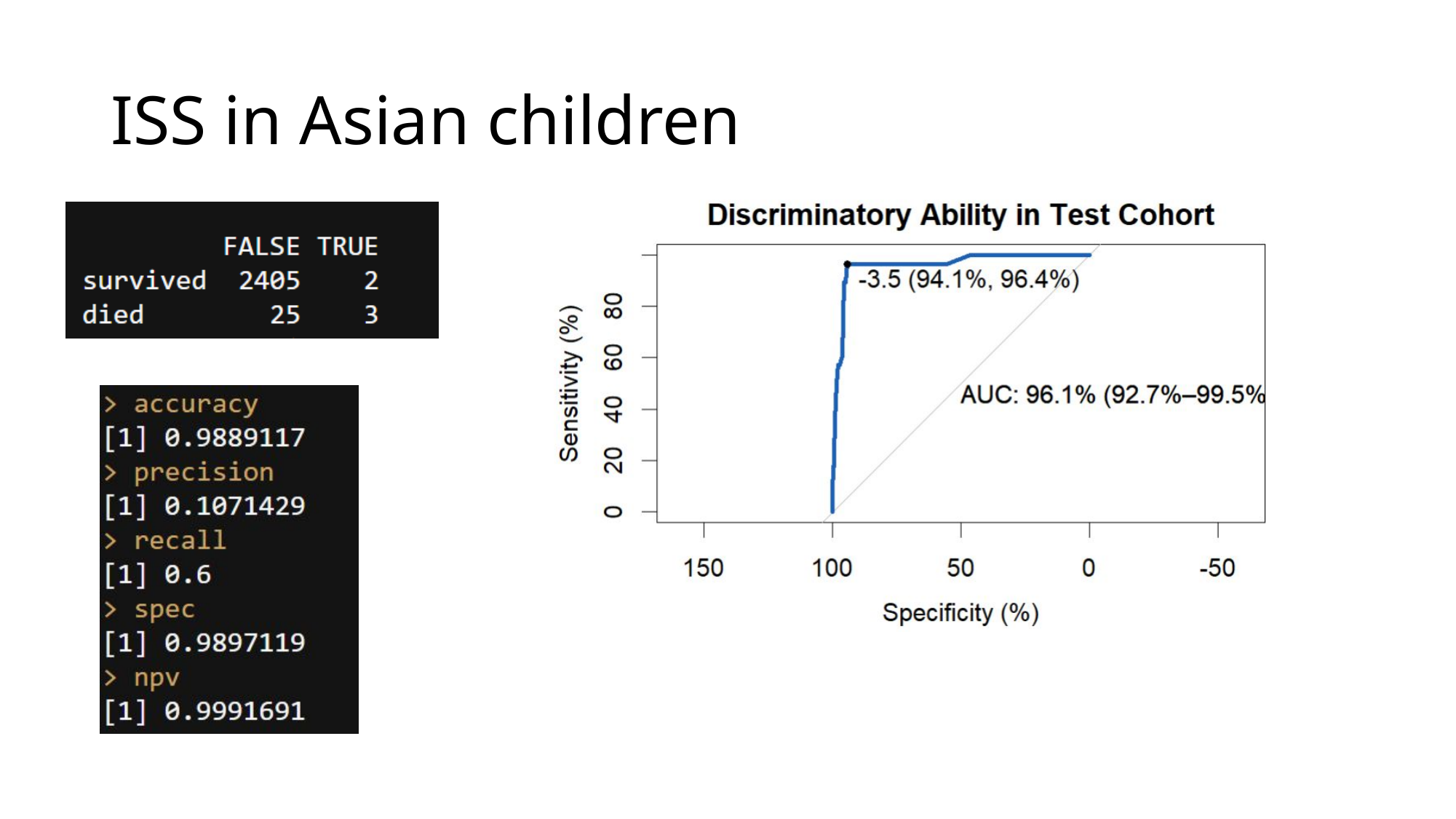

# ISS in Asian children

## Slide 5
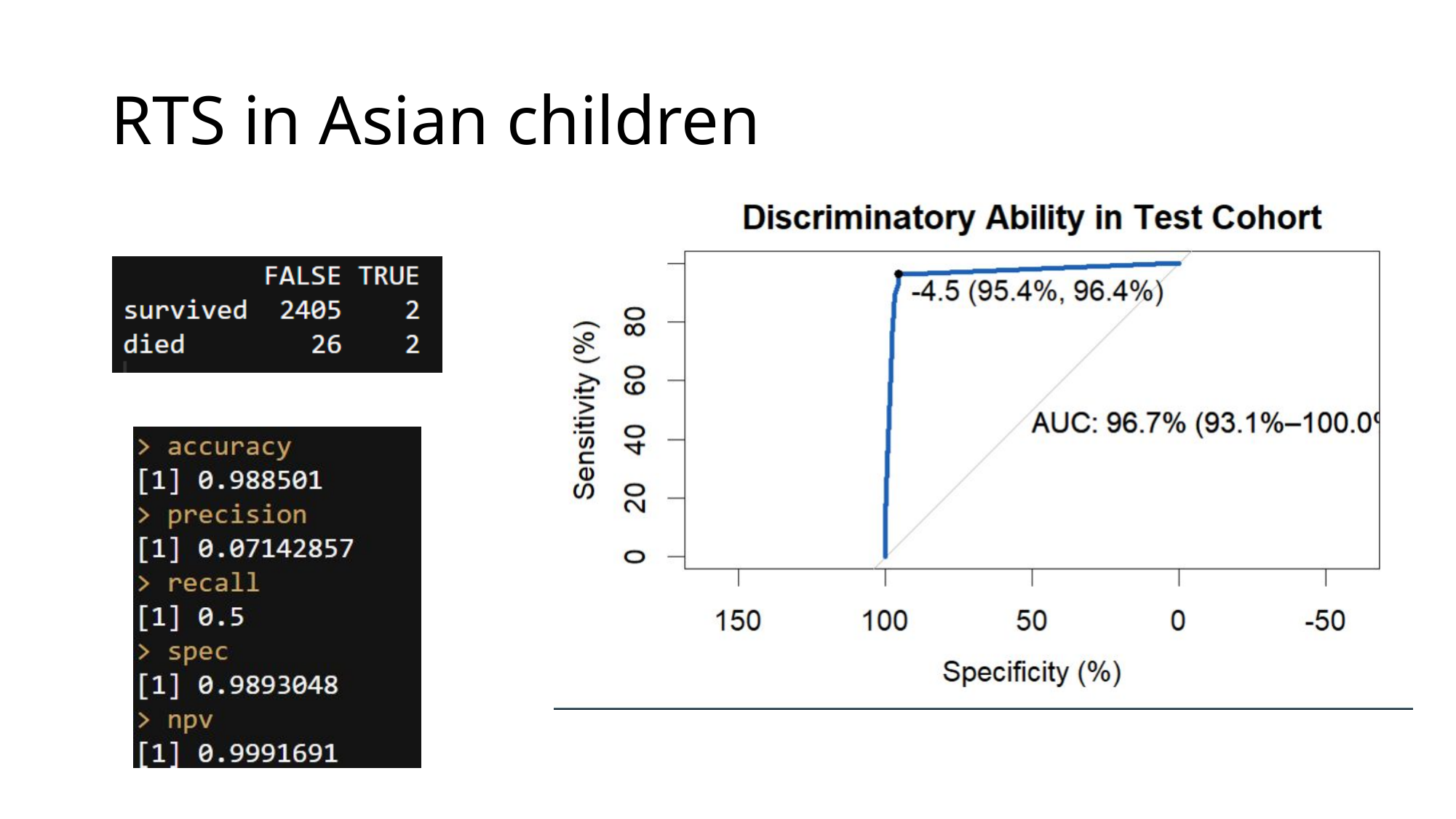

# RTS in Asian children

## Slide 6
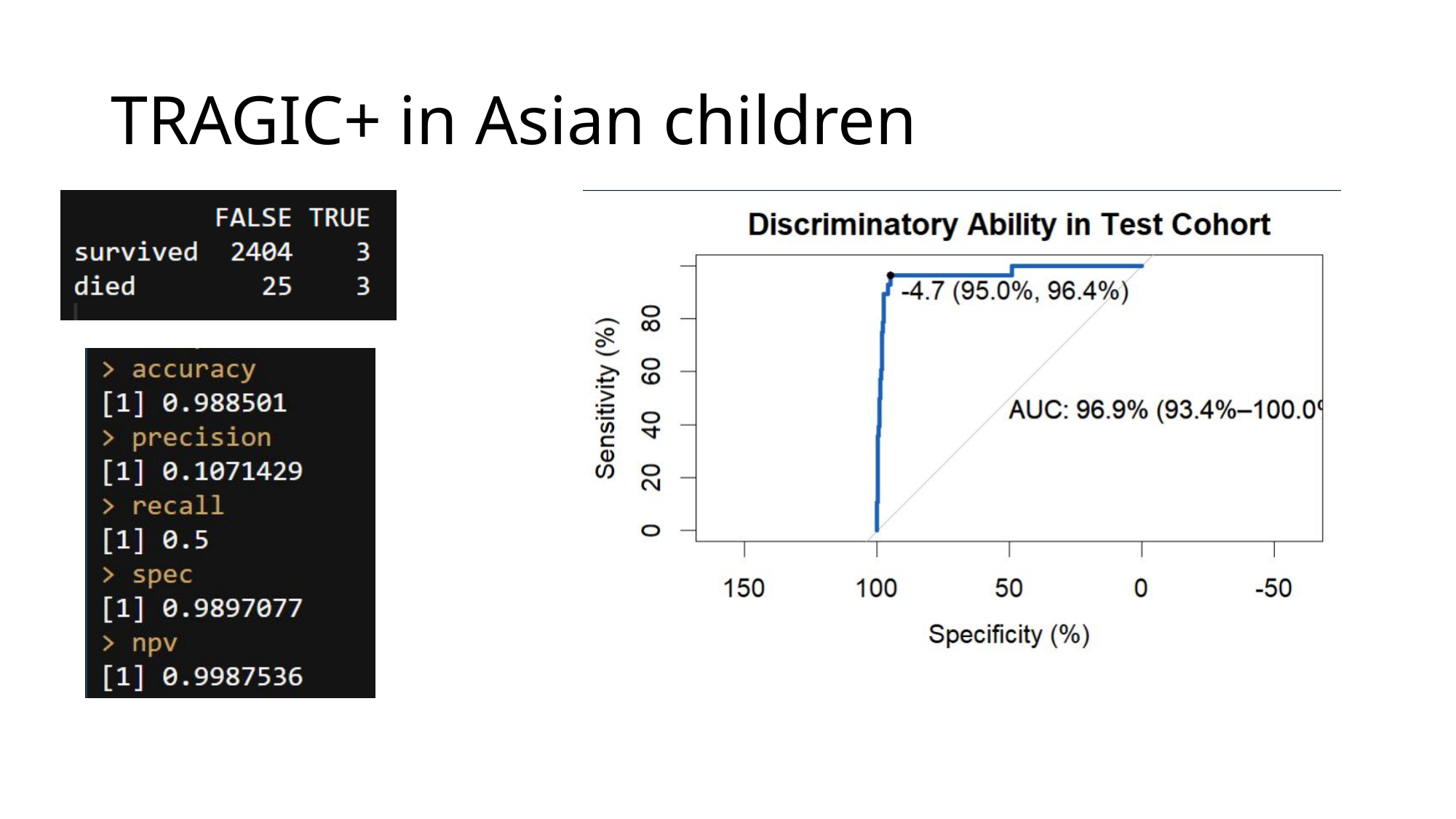

# TRAGIC+ in Asian children

## Slide 7
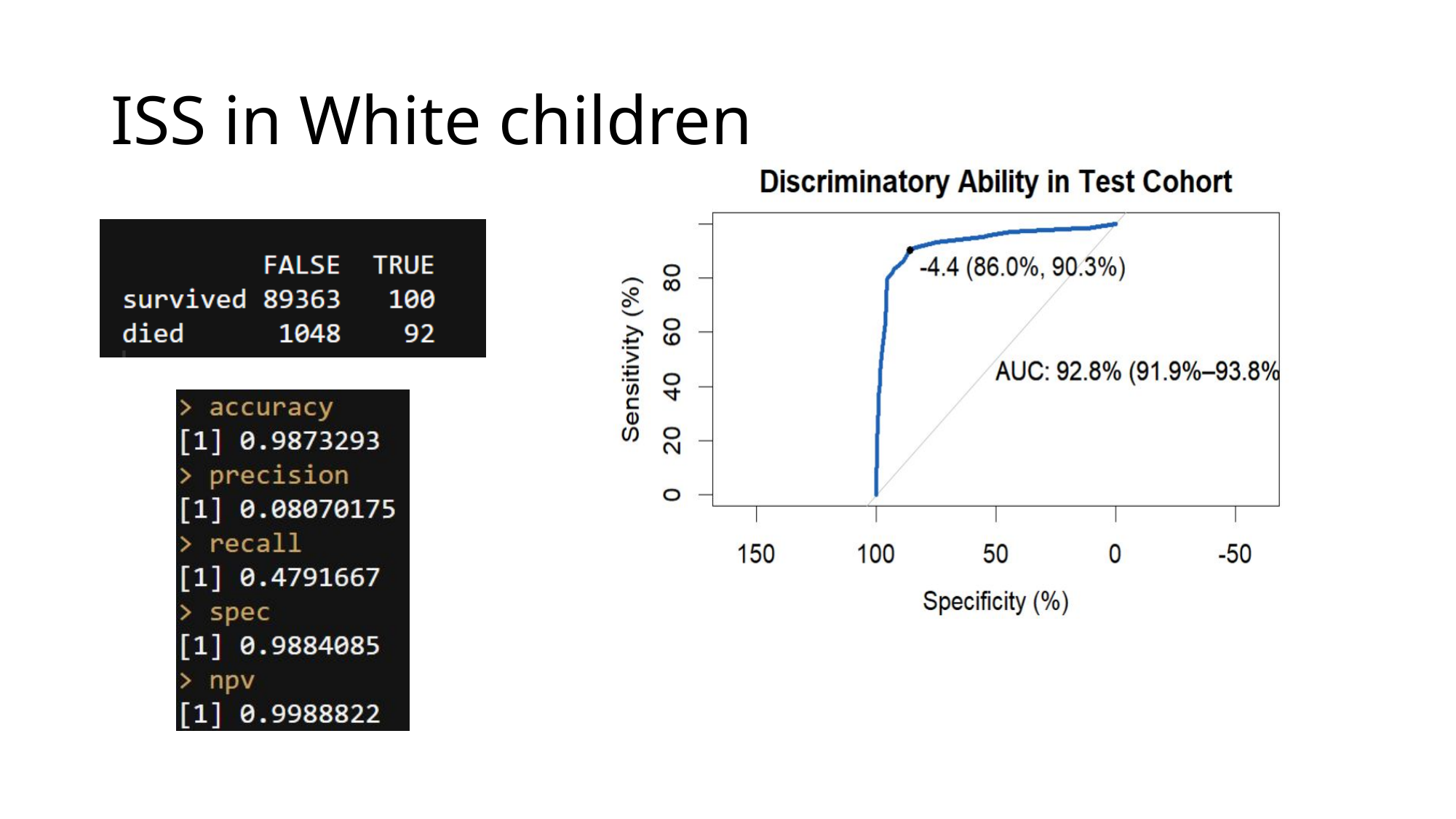

# ISS in White children

## Slide 8
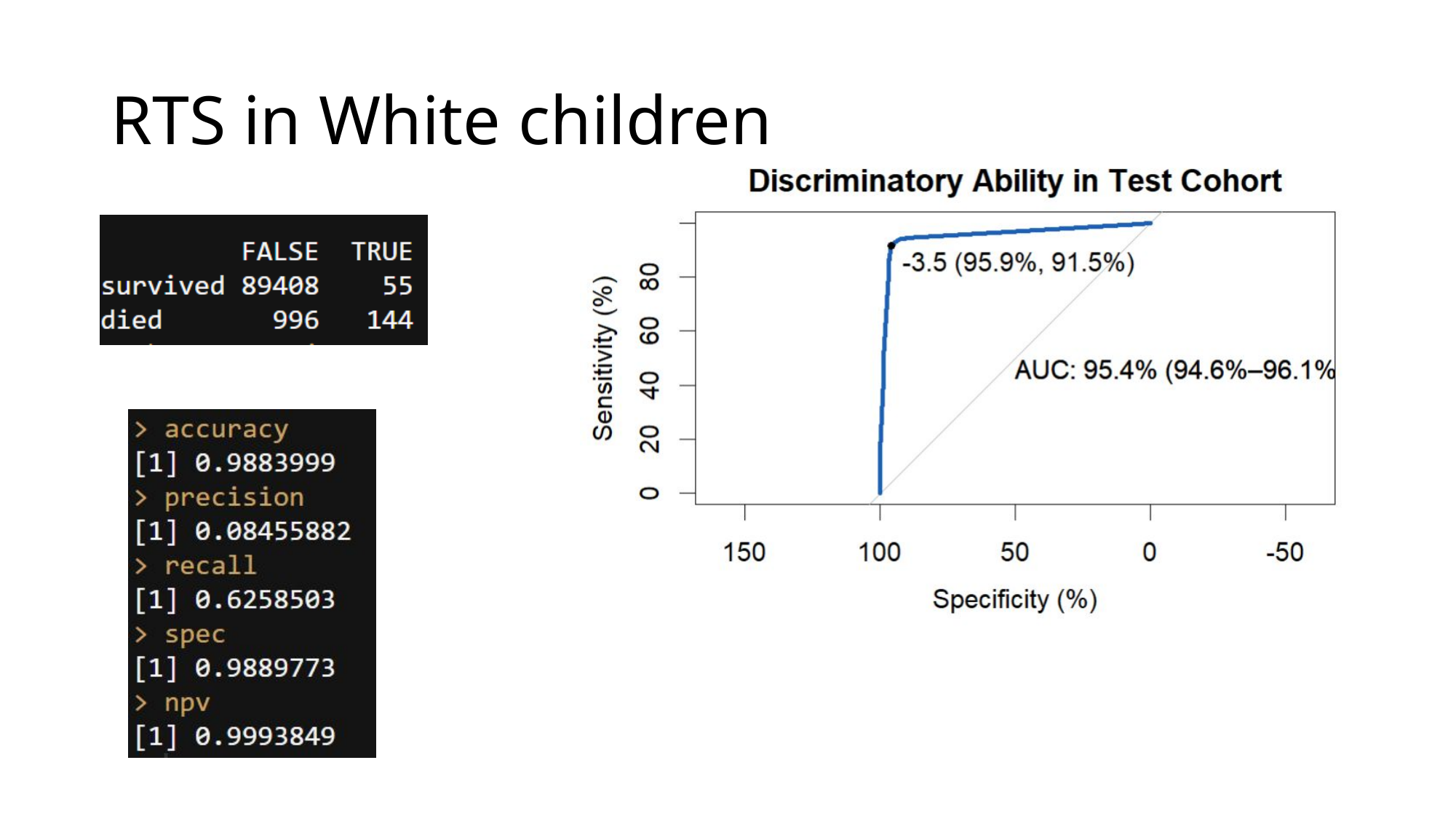

# RTS in White children

## Slide 9
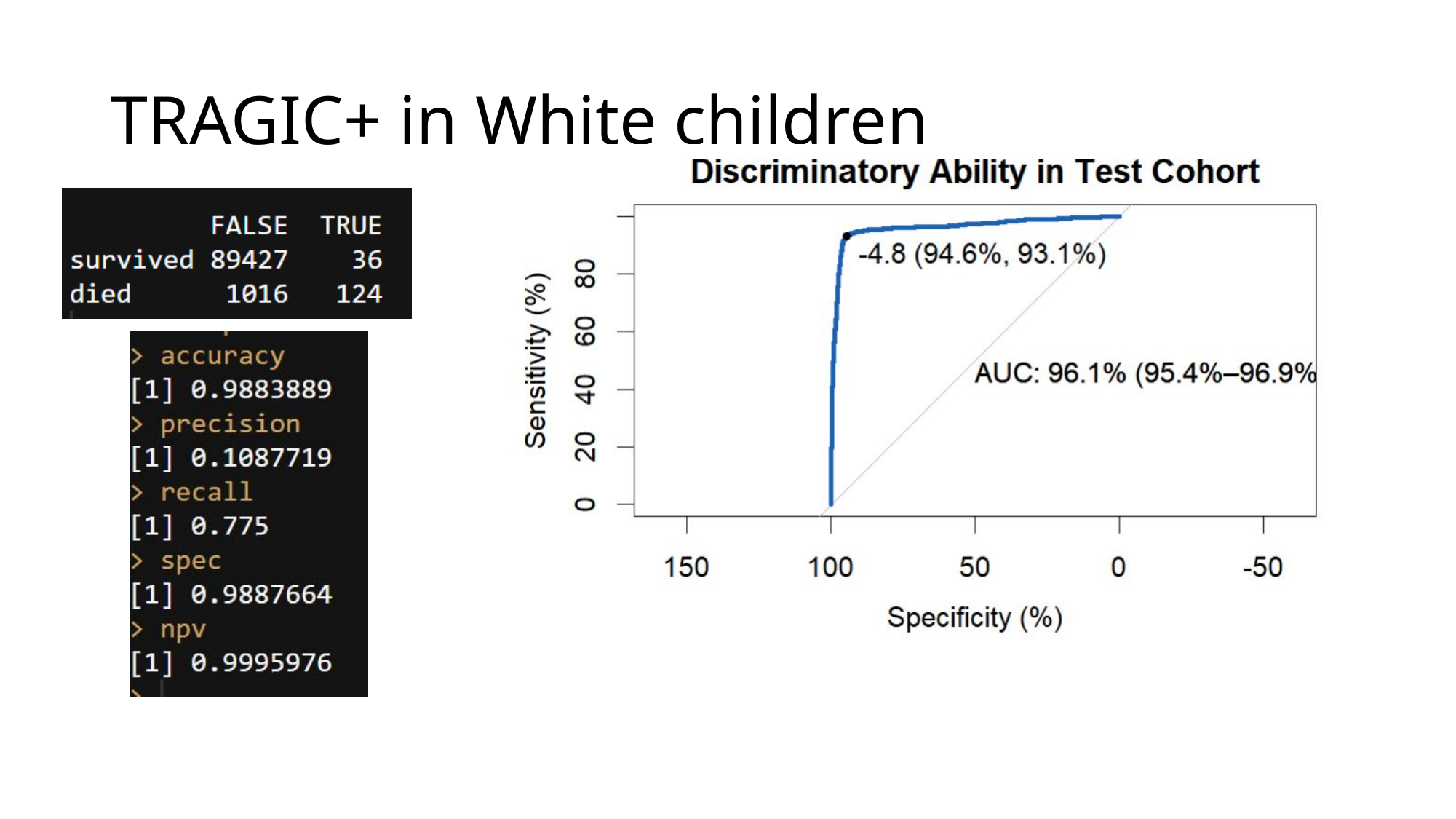

# TRAGIC+ in White children
